# Variations in the Morphology, Mechanics and Adhesion of Persister and Resister *E. coli* Cells in Response to Ampicillin: AFM Study

**DOI:** 10.3390/antibiotics9050235

**Published:** 2020-05-07

**Authors:** Samuel C. Uzoechi, Nehal I. Abu-Lail

**Affiliations:** 1 Department of Biomedical Technology, Federal University of Technology, Owerri PMB 1526, Nigeria; samuel.uzoechi@futo.edu.ng; 2 Gene and Linda Voiland School of Chemical Engineering and Bioengineering, Washington State University, Pullman, WA 99164-6515, USA; 3 Department of Biomedical Engineering and Chemical Engineering, University of Texas at San Antonio, San Antonio, TX 78249, USA

**Keywords:** adhesion, AFM, antibiotics, biopolymers, conformations, *E. coli*, elasticity, multidrug resistance (MDR), persister cells, morphology, resistant cells, roughness

## Abstract

Persister bacterial cells are great at surviving antibiotics. The phenotypic means by which they do that are underexplored. As such, atomic force microscope (AFM) was used to quantify the contributions of the surface properties of the outer membrane of multidrug resistance (MDR)-*Escherichia coli* Strains (A5 and A9) in the presence of ampicillin at minimum inhibitory concentration (MIC) (resistant cells) and at 20× MIC (persistent cells). The properties quantified were morphology, root mean square (RMS) roughness, adhesion, elasticity, and bacterial surface biopolymers’ thickness and grafting density. Compared to untreated cells, persister cells of *E. coli* A5 increased their RMS, adhesion, apparent grafting density, and elasticity by 1.2, 3.4, 2.0, and 3.3 folds, respectively, and decreased their surface area and brush thickness by 1.3 and 1.2 folds, respectively. Similarly, compared to untreated cells, persister cells of *E. coli* A9 increased their RMS, adhesion and elasticity by 1.6, 4.4, and 4.5 folds, respectively; decreased their surface area and brush thickness by 1.4 and 1.6 folds, respectively; and did not change their grafting densities. Our results indicate that resistant and persistent *E. coli* A5 cells battled ampicillin by decreasing their size and going through dormancy. The resistant *E. coli* A9 cells resisted ampicillin through elongation, increased surface area, and adhesion. In contrast, the persistent *E. coli* A9 cells resisted ampicillin through increased roughness, increased surface biopolymers’ grafting densities, increased cellular elasticities, and decreased surface areas. Mechanistic insights into how the resistant and persistent *E. coli* cells respond to ampicillin’s treatment are instrumental to guide design efforts exploring the development of new antibiotics or renovating the existing antibiotics that may kill persistent bacteria by combining more than one mechanism of action.

## 1. Introduction

Multidrug-resistance (MDR) is acknowledged as a global health threat [1,2]. According to the US Centers for Disease Control and Prevention (CDC), two million people experience antibiotic-resistant infections annually and more than 23,000 people die because of such infections [1]. Bacteria can develop resistance to antibiotics through various mechanisms such as the modification of antibiotic target sites on the bacterial surface, inactivation of antibiotics through the production of β-lactamase or similar enzymes, increase in cell wall impermeability to antibiotics, development of efflux pump at the bacterial cytoplasmic membrane to move antibiotics out of cell, and strong adhesion and biofilm formation to impede antibiotic diffusion within cells and biofilms [3,4,5,6].

In addition to the above mechanisms, bacterial resistance to antibiotics can be traced to their abilities to form persister cells [7,8]. Bacterial persistence describes the ability of bacterial cells to get into a dormant, metabolically inactive state, to evade antibiotic stress [9,10]. This phenomenon is exhibited by only ~1% of a given bacterial population. Persister cells were discovered by Hobby et al. in 1942 [10]. Hobby et al. showed that penicillin was not able to kill metabolically non-growing *Staphylococcus aureus* created through reduction of culture temperature [10]. Hobby et al. concluded that the action of penicillin appears to be effective only when the cells are multiplying [10]. By growing *Staphylococcus pyogenes* in a non-nutritive medium, Bigger confirmed that the small population of cells that is “metabolically dormant” and non-dividing survived the effects of penicillin [9]. These cells developed persistence by entering into a physiological dormant state in the presence of stresses such as antibiotics [7,8,9,11,12]. This dormancy has been claimed to be partially responsible for challenges associated with eradicating biofilm infections associated with persister cells [7,8].

Many studies investigated the mechanisms of antibiotic resistance of persister cells in biofilms [8,13,14,15,16,17,18,19]. To quantify eradication rates of persister cells by antibiotics, growth rates of cells were quantified for bacteria grown using nutrient rich or nutrient deprived media [12,20,21]. The presence of nutrients affected the abilities of persister cells to form biofilms. The heterogeneity in the distribution of cells within the biofilm allowed for local microenvironments that vary in the concentration of metabolites, oxygen, waste products and signaling compounds to exist [22,23,24]. Microscopic studies showed evidence of how cells residing within such local microenvironments in the biofilms varied in their metabolic pathways and means of antibiotic tolerance [23,25]. For example, cells within the periphery of nutrients consumed favorable substrates more than cells growing inside the biofilm core; allowing them to form stronger biofilms that were more resistant to antibiotics [23,24]. These studies suggest that nutrient gradients mediate the survival and creation of persister cells in biofilms [23,24].

Furthermore, some studies unveiled genetic basis for the formation of persister cells and, subsequently, their underlying mechanisms of multidrug resistance [26,27]. Genetic basis of persister cells’ tolerance to antibiotics dates back to 1983 when high persistence protein A (*hipA*) gene mutations were discovered in *E. coli* [26]. Recent studies showed that *hipA* encodes the toxin of type II hipAB toxin-antitoxin (TA) locus [27,28]. High persistence protein B (HipB) is the corresponding antitoxin to HipA [27,28]. HipA is generally believed to interrupt the translation of mRNA via phosphorylation and efficiently inhibits cell growth thereby provoking antibiotic resistance [29]. Evidence suggests that bacterial Strains carrying the hipA7 allele produce persister cells at a frequency of ~1% when exposed to ampicillin [30].

In addition to genetic means of persistence to antibiotics, it is important to explore the phenotypic physical mechanisms employed by persister cells to resist antibiotics. These mechanisms reflect contributions of bacterial cell morphology, roughness, adhesion, elasticity, and conformational properties of bacterial surface biopolymers to persister cells’ means of MDR development. Studies in the literature that explored the roles of physiochemical properties of persister bacterial cells on MDR are largely lacking. Without such fundamental knowledge, our ability to guide design efforts aimed at developing effective antibiotics will be hindered. Previously, we explored how resistant Strains of MDR *E. coli* change their physiochemical properties in response to ampicillin at MIC [31,32]. We extended our investigation to explore how persister cells respond to ampicillin at a much higher ampicillin concentration (20× MIC) for a relatively long exposure period (25 h). We hypothesized that persister cells will resist ampicillin through collapsing their surface biopolymers to minimize their interactions with antibiotics as well as to increase their membrane rigidity and impermeability to antibiotics. To test our hypothesis, we used AFM to study changes in bacterial morphology, roughness, adhesion, elasticity, and conformational properties of the persister *E. coli* bacterial surface biopolymers upon exposure to ampicillin.

## 2. Results and Discussion

### 2.1. Effect of Ampicillin Concentration and Exposure Time on Bacterial Viability

**Figure 1:**
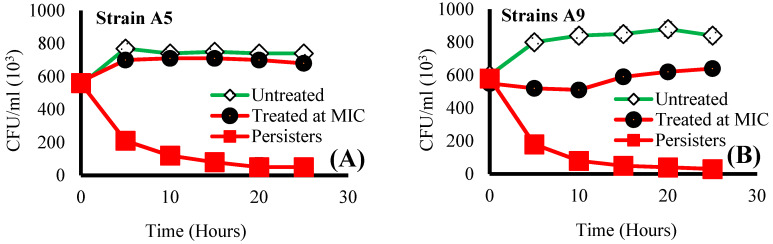
Growth curves of Strains (**A**) A5 and (**B**) A9 as a function of ampicillin treatment. Strains A5 and A9 started with 56 × 10^3^ and 58 × 10^3^ CFU/mL of viable colonies, respectively. Error bars represent the standard error of the means from a three-biological replicate.

Cells representative of both Strains (A5 and A9) reached a plateau in growth within 5 h when untreated (Figure 1). Exposure to ampicillin for 25 h at MIC decreased cell viability (CFU/mL) by 1.1 (5%) and 1.4 folds (29%) for cells of Strains A5 and A9 when compared to untreated cells, respectively (Figure 1). The above indicates that Strain A9 cells were more sensitive to ampicillin at MIC compared to cells of Strain A5 (Figure 1). When compared to untreated, exposure to ampicillin at 20× MIC for 25 h decreased cell viability (CFU/mL) by 4.0 (75%) and 5.0 folds (80%) for cells of Strains A5 and A9, respectively (*p* < 0.001, Figure 1). Percentage survival of persister cells after 25 h exposure to ampicillin at 20× MIC was ~ 9% for Strain A5 and ~5% for Strain A9 (Figure S1). Note that the percentages survival of persister cells reported in this present study are higher than 1% previously reported in the literature [30,33]. These higher survival percentages suggest that cells may even tolerate more than 20-fold MIC of ampicillin. This could be because we isolated our persister cells starting with resistant cell populations while the studies referenced above isolated their persister cells starting with susceptible cell populations [30,33]. The drastic reduction in cell viability for persister and resister cells compared to untreated cells was associated with cellular membrane damage, as can be seen from the SEM images shown later.

### 2.2. Effect of Ampicillin on Bacterial Morphology and Dimensions as Probed by AFM and SEM

**Figure 2:**
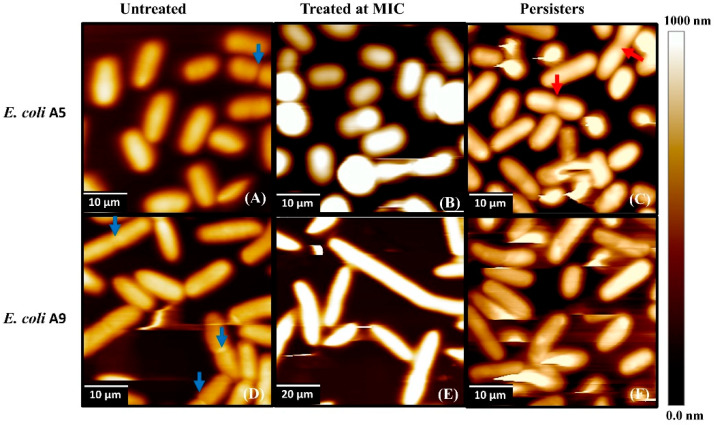
Representative AFM height images showing morphological differences among: (**A**,**D**) natural MDR cells of Strains A5 and A9, respectively; (**B**,**E**) cells treated with ampicillin at various MICs for Strains A5 and A9, respectively; and (**C**,**F**) persister cells of Strains A5 and A9, respectively. Red arrowheads indicate dividing cells in the persister cells’ subpopulations (image **C**), blue arrowheads depict dividing cells in the absence of ampicillin (images **A** and **D**), and green arrowheads show elongated cells after exposure to MIC (image **E**).

AFM height images of bacterial cells captured in water with tapping mode are shown in Figure 2. In all images and irrespective of the treatment condition, bacterial cells were close to each other and each bacterium was clearly distinguished from others in the same image (Figure 2). All cells appeared rod-like within the AFM tip resolution. Exposure to ampicillin for 3 h induced elongation in Strain A9, consistent with our prior findings [31] (Figure 2E). Cells of Strain A9 treated at MIC were 4.2-fold significantly longer than untreated cells (Table S1, *p* < 0.001). The average length of persister cells decreased by 1.2 folds for both Strains when compared to untreated cells (*p* < 0.001). Similarly, when compared to cells treated at MIC, the average length of persister cells decreased by 1.1 and 4.8 folds for Strains A5 and A9, respectively (*p* < 0.001, Table S1). Cells treated at MIC were significantly longer when compared to persister cells and untreated cells for both Strains (Table S1, *p* < 0.001). In addition, the widths of persister cells for both Strains were significantly smaller by 1.3 and 1.2 folds when compared to untreated, and by 1.2 and 1.8 folds when compared to cells treated at MIC, respectively (Table S1, *p* < 0.001). As shown in Figure 2, cells were swollen and resolving their ultrastructure and surface features such as their surface asperities can be very challenging [34].

**Figure 3:**
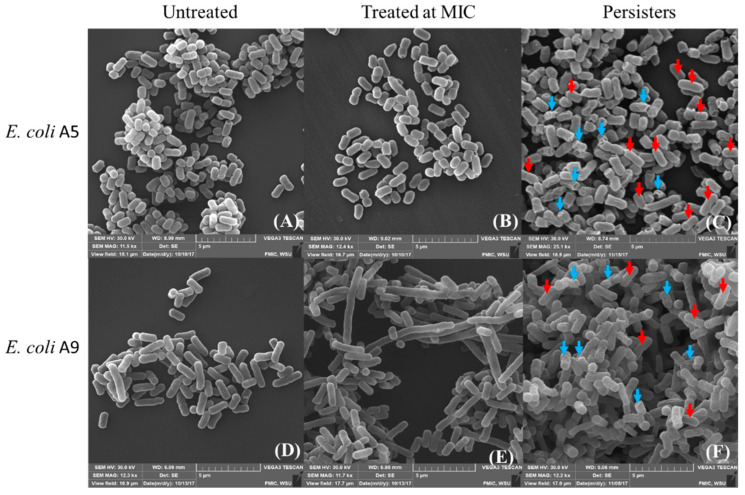
Representative SEM images of: (**A**,**D**) MDR *E. coli* untreated cells of Strains A5 and A9, respectively; (**B**,**E**) cells of Strains A5 and A9 exposed to ampicillin at MIC for 3 h, respectively; and (**C**,**F**) persister cells of Strains A5 and A9 exposed to ampicillin at 20× MIC, respectively. Red arrows on images **C** and **F** for both Strains show intact cells with expected surface morphologies, while blue arrows on images **C** and **F** indicate cells with ampicillin-induced damage, *n* = 3 independent cultures. Note the elongation of A9 cells when treated at MIC (image **E**).

To investigate what happens to the cells’ ultrastructures, we turned our attention to high-resolution images acquired with SEM (Figure 3). The SEM images clearly depicted the expected elongation of Strain A9 cells as a result of exposure to ampicillin at MIC (Figure 3F). The results of SEM imaging are consistent with those of AFM height images (Figure 2). The SEM images depicted a portion of persister cells with a normal elliptical surface morphology that is intact for both Strains investigated and a portion of cells with membrane damage due to the treatment with ampicillin (Figure 3). Even though the SEM images provided qualitative representation of the ultrastructure of the bacterial surfaces, caution should be used in interpreting such images due to the harsh and multiple sample preparations required prior to imaging the cells [35,36].

The AFM and SEM images (Figure 2 and Figure 3) suggest that at MIC, treatment with ampicillin did not disrupt the A5 cell membrane. Consistent with AFM and SEM images, we showed previously using fluorescence imaging that cells of both Strains A5 and A9 do not lyse their cell membranes or release their DNA content when treated with ampicillin at MIC for 3 h [31]. The morphologies of A5 resistant and persistent cells were always elliptical and slightly smaller in surface area as well as in surface area to volume ratio compared to those of untreated cells (Figure 2 and Figure 3 and Table S1). The reduction in cellular dimensions suggests that cells are adopting dormancy as a survival mechanism through which they conserve their energy [37]. When cells of A9 were considered, despite elongation being observed when cells were treated at MIC and shrinkage when cells were treated at 20× MIC, the bacterial membrane integrity did not seem to be affected in all conditions (Figure 2). Cells of Strain A9 treated at MIC are thought to resist ampicillin through increased contact with surfaces. Such better contact is expected to allow them to form stronger biofilms [31,32]. At 20× MIC, cells might become dormant to conserve their energy in order to resist ampicillin.

### 2.3. Effect of Ampicillin Concentration and Exposure Time on Cellular Surface Area (SA), Volume (V), and Surface Area to Volume Ratio (SA/V)

**Figure 4:**
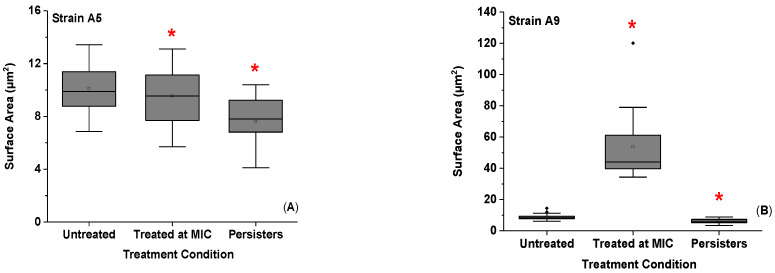

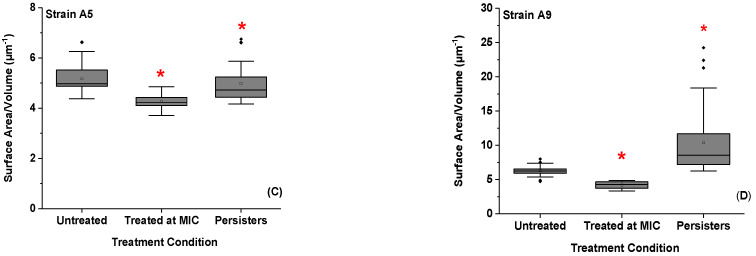
Summary of (**A**,**B**) the average surface area and (**C**,**D**) the surface area to volume ratio for MDR *E. coli* A5 and A9 untreated cells, cells exposed to ampicillin at MICs and persister cells exposed to 20xMIC, respectively. (**A**,**C**) For Strain A5, 45, 30, and 34 cells were probed, respectively, whereas, for Strain A9, 38, 45, and 39 cells were plotted, respectively. * Values are statistically significant between the untreated and treated cells, *p* < 0.001, *n* = 3 independent cultures.

When exposed to ampicillin at MIC for 3 h, the SA of Strain A5 decreased by 1.1 folds while that of Strain A9 increased by 6.3 folds compared to untreated cells (*p* < 0.001, Figure 4A,B). The persister cells differentiated themselves by significant smaller surface areas compared to the cells treated at MIC and untreated cells for both Strains A5 and A9 (*p* < 0.001, Figure 4A,B) leading to an increase in the SA/V (*p* > 0.001, Figure 4C,D). For Strain A5, the SA of the persister cells decreased by 1.3 folds compared to the untreated or treated cells at MIC (*p* < 0.001, Figure 4A,B). The corresponding SA/V increased by 1.0 and 1.2 folds compared to untreated and cells treated at MIC, respectively (*p* < 0.001, Figure 4C). Similarly, the SA of persister cells of Strain A9 decreased by 1.4 and 8.8 folds when compared to untreated cells and cells treated at MIC, respectively (*p* < 0.001, Figure 4D). The corresponding SA/V increased by 1.6 and 2.4 folds compared to untreated cells and to cells treated at MIC, respectively (*p* < 0.001, Figure 4C).

How bacterial cells construct their shapes depends largely on their environments and genetic makeup [38,39,40,41,42]. It is assumed that upon exposure to stimuli, many bacterial species keep their shapes unchanged while varying their volumes [39]. For example, the volumes of *E. coli* can increase or decrease based on the ionic strength of the media they grow in while their shapes remain elliptical in all media investigated [39]. Changes in volume can lead to changes to the surface area to volume ratio [39]. The above held true for our study. Cells of both Strains maintained always elliptical geometries upon exposure to ampicillin for different times and at different concentrations. However, their volumes and surface areas were quite dependent on the treatment conditions used (Table S1 and Figure 4).

Studies in the literature investigated how bacterial cells change their dimensions in response to nutrient starvation [43], genetic modifications [44], and antibiotics [41]. Even though these studies were not done on persister cells, findings from them are helpful for us to explain some of our findings. For example, it is well accepted that bacterial cells increase their length just before they divide [42,45]. Furthermore, because the time needed to replicate the chromosomes as well as the time required for cells to divide is constant, it is expected that faster-growing cells elongate and become more rod-like than slow-growing cells [42,45]. This phenomenon could explain the variations in cell lengths observed between the persister cells and resistant cells when compared to untreated cells for both strains (Table S1). The shorter cells in our study include persister cells of both Strains and resistant A5 cells. All these cells have lengths that are below the lengths of their counter untreated cells. Being short may be the result of slow DNA replication in these cells [42]. Exposure to ampicillin at 20× MIC for 25 h decreased cell viability (CFU/mL) by 4.0 (75%) and 5.0 folds (80%) for Strains A5 and A9, respectively (Figure 1). This means that these cells are possibly slow growers. Antibiotics corrupt metabolic processes in rapidly growing cells and induce cell death. This is why keeping the cells short might be a mechanism employed by cells to minimize the effects of antibiotics on their metabolism [7,11].

On the contrary, this phenomenon cannot be used to describe the elongation mechanism observed for resistant A9 cells. Our results indicate that, at this treatment concentration and time, the growth of cells of Strain A9 was inhibited, as evident from the 230 × 10^3^ CFU’s/mL lower viable colonies than that quantified for untreated cells (Figure 1). This finding supports studies that showed that antibiotics corrupt metabolic processes in rapidly growing cells and induce cell death [19,46]. Therefore, cells of Strain A9 may benefit temporarily from their elongation when exposed to ampicillin at MIC by increasing their surface areas to enhance adhesion to potential surfaces and to form biofilms that can survive the harsh treatment with antibiotics [31]. However, as the elongation come with its own drawbacks, cells attempt to recover their short dimensions with longer exposure to antibiotics (8 h in our previous studies [31,32] and 25 h here).

### 2.4. Effect of Ampicillin Concentration and Exposure Time on Bacterial Surface Roughness

**Figure 5:**
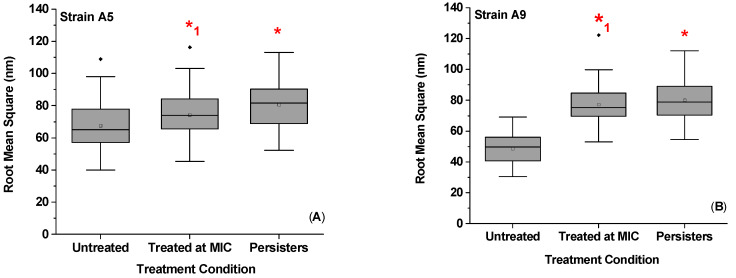
The average bacterial surface RMS in nm obtained on 0.60 × 0.60 µm^2^ areas of surfaces of cells that belong to Strain A5 (**A**) and Strain A9 (**B**) for variable treatments. For Strain A5, 45, 30, and 34 cells were probed, respectively, whereas, for Strain A9, 38, 45, and 39 cells were plotted, respectively. * Values are statistically significant between untreated and treated cells. *^1^ Values are statistically significant from untreated cells but not from the treated groups, *p* < 0.001, *n* = 3 independent cultures.

Higher RMS roughness values were found for persister cells when compared to untreated cells. Specifically, RMS values were 1.2-fold (16%) and 1.6-fold (39%) higher in Strains A5 and A9 persister cells compared to untreated cells (*p* < 0.001, Figure 5). When RMS values of persister cells were compared to RMS values of cells treated at MIC, no significant differences were observed for both Strains (*p* > 0.001, Figure 5). The RMS of cells treated at MIC increased by 1.1 (9%, *p* > 0.001) and 1.6 folds (37%, *p* < 0.001) compared to untreated cells with no visible cell damage or shrinkage of cells’ volumes (Figure 2, Figure 3 and Figure 5). In comparison, the increased RMS values observed for persister cells were associated with membrane roughness and shrinkage of cells (Figure 3).

Exposure to ampicillin increased the cell surface roughness in a concentration and time-dependent manners (Figure 5). Consistent with our observations summarized above, an increase in the cell surface roughness as a result of treatment with antimicrobial peptides [47,48] and ampicillin [49] have been previously reported for *E. coli* susceptible cells. These studies showed signs of cell membrane destruction. Here, our AFM and SEM images suggest no signs of membrane damage or leakages of membrane content (Figure 2 and Figure 3C,F). This is likely because we are working with resistant cells not susceptible cells. The higher cell surface roughness observed here may originate from increased expression of different biopolymers on the bacterial surface [41]. Increasing the diversity of bacterial surface molecules can be utilized as a survival strategy by bacterial cells exposed to stress. This strategy was evident here as we can see that all properties estimated for the bacterial cells of both Strains (adhesion force, biopolymer brush thickness, grafting density, and elasticity) displayed heterogeneities (Figures S2–S5). Variable biopolymers differ in their side groups, charges, and wettabilities and, as such, can support variable bacterial functions. For example, having a heterogeneous population of bacterial surface biopolymers has been shown to correlate positively with higher adhesion energies [50,51]. Higher initial attachment of bacterial cells to surfaces promote biofilm formation [52,53,54,55]. Literature studies have shown that, the rougher is the surface of bacteria, the easier it is for cells to attach firmly to a surface [56]. A rougher bacterial surface has also been shown to promote attractive van der Waals forces which can be a mean employed by bacterial cells to enhance their adhesion to surfaces [57]. Increase in bacterial expression of surface molecules is expected to result in an increase of the grafting density of a bacterial surface biopolymeric brush. This indeed was the case here. The bacterial surface roughness was directly proportional to the grafting density estimated for the bacterial surface (Figure S6D). More molecules are associated with higher adhesion as well. Finally, bacterial surface biopolymers are also important in keeping the bacterial membrane integrity [58]. The more intact are the bacterial surfaces, the harder it is for antibiotics to invade them.

### 2.5. Effect of Ampicillin Concentration and Exposure Time on Adhesion Forces Measured between the Biopolymers of MDR E. coli Cells and a Model Surface of Si_3_N_4_

**Figure 6:**
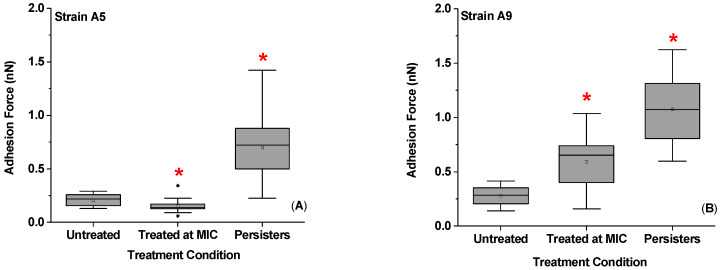
A summary of the average AFM nanoscale adhesion forces (F_ad_) measured between the Si_3_N_4_ cantilever and the bacterial surface biopolymers: (**A**) for A5; and (**B**) for A9. (**A**) For Strain A5, 816, 260, and 273 adhesion point were plotted, respectively. (**B**) For Strain A9 the average of 205, 91, and 41 adhesion points were plotted, respectively. * Values are statistically significant from the control and treatment group at *p* < 0.001, *n* = 3 independent cultures.

A summary of the adhesion forces measured between bacterial surface biopolymers and Si_3_N_4_ as a function of ampicillin is shown in Figure 6 and Figure S2. Exposure to ampicillin at MIC significantly decreased the average adhesion force for A5 cells to Si_3_N_4_ by 1.3 folds compared to untreated cells (23%, *p* < 0.001) (Figure 6A). On the contrary, treatment of cells of Strain A9 at MIC significantly increased the average adhesion force measured between the bacterial surface biopolymers and Si_3_N_4_ by 2.3 folds (57%, *p* < 0.001). When persister cells were considered, 25 h of treatment with ampicillin at 20× MIC increased the average adhesion force in both Strains by 3.4 (70%) and 4.4 folds (77%) when compared to the untreated, respectively (*p* < 0.001, Figure 6). When cells exposed to ampicillin at MIC were compared to persister cells, the average adhesion force in persister cells was higher by 4.4 (77%) and 1.9 folds (47%) for Strains A5 and A9, respectively (*p* < 0.001, Figure 6). This increase in adhesion forces between persister cells’ biopolymers and Si_3_N_4_ is consistent with an increase in bacterial surface roughness for the persister cells (Figure 5 and Figure 6).

Cellular adhesion to surfaces depends on many factors. Our data suggest that adhesion was only correlated with the grafting density of the bacterial surface biopolymer brush (Figure S6C). Other factors including biopolymer brush thickness, roughness, and surface area were not as correlated to adhesion (Figures S6 and S7). Furthermore, our data indicate that adhesion increased for treated MDR and persister cells compared to untreated cell except for Strain A5, for which cells treated at MIC had lower adhesion than untreated cells (Figure 6). Bacterial adhesion to surfaces is a complex phenomenon and constitutes the main mechanism employed by bacterial cells for variable functions including access to nutrients, building a biofilm community and having an improved antibiotics’ resistance [53,54].

Previous AFM studies investigating bacterial adhesion changes in response to ampicillin demonstrated an increase in adhesion that was positively correlated with increased surface roughness for both susceptible *B. subtilis* and *E. coli* cells treated with ampicillin [49]. A higher adhesion was also observed for susceptible *P. aeruginosa* cells grown in a biofilm culture in the presence of colistin [59]. The authors attributed the increase in adhesion after treatment with antibiotics and antimicrobial peptides to the loss of cell internal content [49,59]. Colistin displaces the Ca^2+^ and Mg^2+^ from the membrane lipid phosphate groups present on the outer bacterial membrane resulting in a membrane disruption and leakage of cells’ internal content [60]. This mechanism is similar to that employed by ampicillin. Ampicillin inhibits the PG synthesis by interfering with PBPs leading to cell lysis [61,62]. For the work presented by San-Serramitjana et al. and Laskowski et al., it is likely that the disruption of the cell wall allowed the AFM tip to penetrate inside the cell cytoplasm resulting in contacting more biomolecules that enhanced adhesion forces [49,59]. Ampicillin is known to induce a disruption in the membranes of susceptible cells and that may result in increased adhesion [49,59]. In our work and within the AFM resolution, it appears that ampicillin treatment at MIC or at 20× MIC did not disrupt the membranes of cells. Therefore, the increased adhesion observed here for the MDR *E. coli* and persister cells of Strain A5 and the MDR cells of Strain A9 is likely due to the increase in the biopolymers’ grafting densities and expression of diverse population of biopolymers.

Another strategy the bacterial cells may use to increase their surface adhesion to the Si_3_N_4_ upon ampicillin treatment is through surface modifications of their hydrophobicities [49]. In our previous study, we demonstrated that exposure to ampicillin enhanced the hydrophilicity of Strain A9 while made Strain A5 slightly more hydrophobic [31]. For Strain A9, masking their hydrophobic PG layer completely may result in higher adhesion to the hydrophilic Si_3_N_4_ tip [49]. This mechanism is important in hindering the ampicillin from reaching the PG [31]. Since Si_3_N_4_ is a hydrophilic substrate in water [63], ampicillin is also hydrophilic [64]. The increased adhesion observed for cells exposed to antibiotics requires increased hydrophilicity, which was the case here for A9 [31]. We showed previously that the surface interactions of Strains A5 to Si_3_N_4_ were dominated by electron acceptor (γ_s_^+^) and a very low electron donor (γ_s_^−^) surface energies in the presence and absence of ampicillin, respectively [31]. However, it is important to note that a high surface energy of the bacterial cell wall does not always guarantee a high cellular adhesion or biofilm formation to a hydrophilic surface [65]. In the future, wettabilities of persister cells should be quantified to detail the roles of acid–base forces in governing how persister cells interact with surfaces.

### 2.6. Variations in the Thicknesses and Grafting Densities of Bacterial Surface Biopolymer Brushes in Response to Ampicillin at Different Concentrations and Times

**Figure 7:**
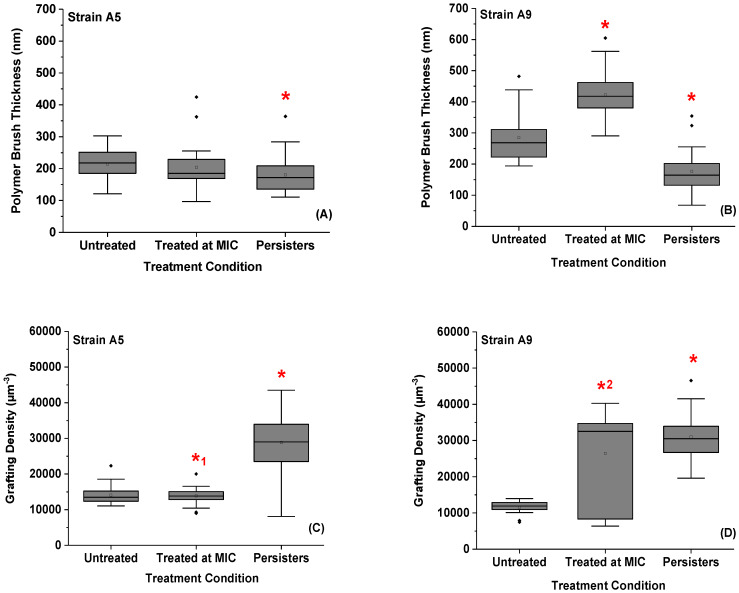
Boxplots showing the distributions of biopolymer brush thicknesses (**A**,**B**) and grafting densities (**C**,**D**) of the MDR *E. coli* Strains estimated using the steric model, Equation (1), or untreated cells, cells treated at MIC, and persister cells, respectively. * Values are statistically significant between the untreated and treated cells. *^1^ Values are statistically significant from untreated cells but not from the treated groups and *^2^ Values are statistically significant from the treated group but not from the untreated group at *p* < 0.001, *n* = 3 independent cultures.

The thicknesses and grafting densities of bacterial surface biopolymer brushes for both Strains in all conditions ranged from 178 to 403 nm and from 11,426 to 30,871 μm^−2^, respectively (Figure 7, Figures S3 and S4). Upon treatment with ampicillin at MIC for 3 h, the average brush thicknesses for biopolymers of cells of Strains A5 and A9 increased by 1.1 (5%, *p* > 0.001) and 1.4 folds (29%, *p* < 0.001) compared to untreated cells (Figure 7A,B). The increase in these thicknesses was associated with an increase in grafting densities of 2.6 folds (61%, *p* < 0.001) in Strain A9, but did not show any significant differences in Strain A5 (1%, *p* > 0.001) from untreated cells (Figure 7C,D).

The biopolymers of the persister cells collapsed on their surfaces leading to a decrease in their brush thicknesses by 1.2 (15%, *p* < 0.001) and 1.6 folds (38%, *p* < 0.001) when compared with thicknesses of untreated cells’ biopolymer brushes of Strains A5 and A9, respectively (Figure 7C,D). Similarly, the average brush thicknesses of the persister cells decreased by 1.1 (11%) and 2.3 folds (56%) for Strains A5 and A9, respectively, when compared to cells exposed to ampicillin at MIC (*p* < 0.001, Figure 7C,D). The collapse of the biopolymer brushes of the persister cells was associated with a significant increase in their apparent grafting densities as compared to those of untreated cells (*p* < 0.001, Figure 7C,D). The average apparent grafting densities of the persister cells were only significantly different from those treated at MIC in Strain A5 where a 2.0-fold (51%) increase was observed (*p* < 0.001, Figure 7C). The reduction in the average thickness of the surface biopolymer brush observed for persister cells was inversely proportional to adhesion forces (Figure S6A) and to bacterial surface roughness (Figure S6B).

The bacterial surface is decorated with surface biopolymers of lengths that can extend up to hundreds of nanometers into the environment [66]. These biopolymers have different structures and functions, which include inducement of genes that regulate cell shape, facilitating initial attachment and biofilm formation [67]. Because of the many biopolymers that cover the bacterial surface, a range of biopolymer brush thickness (*L*_0_) and grafting density (*Γ*) values are expected [66]. This was indeed the case here (Figures S3 and S4, respectively). Our results indicate that short exposure to ampicillin for 3 h resulted in cells that are more heterogeneous compared to when cells were exposed to ampicillin for longer times [32], suggesting that the longer exposure to ampicillin allowed cells to adapt to the stimuli and that resulted in a more homogenous population of cells.

In this study, we showed that bacterial cells exposed to ampicillin at all-time points and concentrations are decorated with more abundant surface biopolymers and higher adhesion compared to their relative controls (Figure 7). Excretion of more biopolymers upon exposure to ampicillin is expected to provide the MDR and persister cells with an edge for better survival. A stronger attachment to a surface has been shown to stimulate nutrients’ capture especially in the nutrients deficient environments [68]. In addition to nutrient capture, bacterial attachment to a surface stimulates biofilm formation, which is expected to provide extra protection to the bacterial surface against antibiotics [69,70].

### 2.7. Variations in the Elasticities of Bacterial Cells in Response to Ampicillin at Different Concentrations and Times

**Figure 8:**
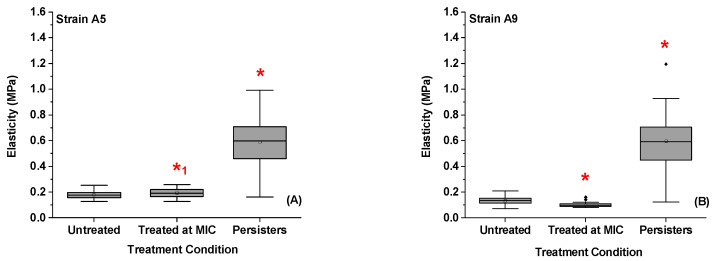
(**A**,**B**) A summary of the average bacterial cell elasticities quantified for Strains A5 and A9, respectively by Hertz model described in Equation (2) for different treatments. * Values are statistically significant between the untreated and treated cells. *^1^ Values are statistically significant from the treated group but not from the untreated group, *p* < 0.001, *n* = 3 independent cultures.

Exposure to ampicillin at MIC decreased the average elasticity of cells of Strain A9 by 1.4 folds (27%, *p* < 0.001) and did not influence the elasticity of cells of Strain A5 (8%, *p* > 0.001) when compared to elasticities of untreated cells (Figure 8A,B and Figure S5). These results are consistent with our previous work [32]. In comparison, the persister cells increased their elasticities by 3.3 (70%) and 4.5 folds (78%) when compared to untreated, and by 3.0 (67%) and 6.1 folds (84%) when compared to cells treated at MIC for A5 and A9, respectively (*p* < 0.001, Figure 8A,B).

The ability of bacteria to resist antibiotics is in part due to changes made to their cell wall composition, conformations of biopolymers, charge, and hydrophobicity, as well as to how such surface changes affect cellular elasticities and enable cellular interactions with the environment [71,72,73]. The bacterial cell wall is known to protect the cell membrane from rupture caused due to the high internal turgor pressure as well as controls the integrity and morphology of the cell [74,75]. The bacterial rigidity quantified in terms of Young’s modulus mirrors the resistance of the bacterial cell wall to mechanical compression and the stiffness of the PG network and other cell wall constituents such as surface biopolymers [76,77,78].

In our previous study, investigating the changes in cell elasticity of MDR *E. coli* cells exposed to ampicillin at MIC at different time points indicated that Strain A9 became elongated and softer at short time exposure to ampicillin to enhance their potentials to increase their surface areas and as a result increase their biofilm formation [31]. We demonstrated that softer cells were associated with extended biopolymer brushes and decreased grafting densities [32]. The longer exposure to ampicillin decreased the brush thickness and increased the grafting density and elasticities of cells of Strain A9 [32]. Unlike A9, short time exposure to ampicillin did not change the elasticity of cells of Strain A5 [32]. Strain A5 did not change its biopolymer conformational properties in response to ampicillin either [32]. These findings indicate that manipulating the cellular mechanics may not be an important means through which Strain A5 responds to antibiotics [32]. Interestingly, the current study revealed that persister cells isolated from Strains A5 and A9 are stiffer than MDR and untreated cells. The increase in the elasticity is directly proportional to the increase in the grafting density (Figure S6F). This increased elasticity possibly reflects increased impermeabilities in cellular membranes and as such is an expected mechanism that allows cells to limit the diffusion of antibiotics to cells. To confirm this speculation, we recommend that permeabilities of cellular membranes to be measured in the future.

Studies in the literature that addressed the effects of antibiotics on cellular mechanics are rare. In one such study, the effects of antibiotics on the cell membrane of resistant *Pseudomonas aeruginosa* demonstrated the ability of ticarcillin to disorganize the cell membrane at 4 mg/mL, resulting in a reduction of resistant Strain elasticity from 500 ± 100 to 300 ± 66 KPa [76]. In another study, Perry et al. demonstrated that Young’s moduli of *E. coli* ATCC 9637 cells decreased from 2.2 ± 0.09 to 1.1 ± 0.13 MPa upon exposure to ampicillin at 25 μg/mL [77]. In a third experiment, Longo et al. measured the mechanical stiffness of susceptible *E. coli* DH5α upon exposure to ampicillin [79]. Growing *E. coli* in LB supplemented with ampicillin below the MIC at 0.18 μg/mL caused dramatic changes in the bacterial morphology ranging from membrane deflation to lysis [79]. The elasticity of the cells was reduced from 300 ± 70 to 100 ± 20 KPa after exposure to ampicillin, indicating softening in the cell membrane [79]. However, we did show in our previous study that some of the MDR Strains decreased their elasticities in response to ampicillin while others increased them. In both circumstances, their membranes remained intact [32]. Here, we showed that longer exposure to ampicillin resulted in stiff persister cells that can protect the cell membrane possibly through limiting high diffusion of antibiotics into the cells.

### 2.8. Possible Mechanisms Employed by MDR E. coli Resistant and Persistent Cells to Resist Ampicillin

Due to their perseverance, many studies have investigated the possible means through which persister cells resist antibiotics at the macroscale [8,14,15,16,17,18,19]. For example, Shal et al. differentiated *E. coli* persister cells resulting from ofloxacin treatment at 5 μg/mL from their wild-type based on their gene expressions [8]. The authors hypothesized that persister cells are slow growing cells with a low level of proteins’ synthesis [8]. By analyzing the cells using fluorescence activated cell sorting (FACS) with green fluorescence protein (GFP), Shah et al. demonstrated that a small subpopulation of cells has no detectable fluorescence and labeled that population as persisters. The bright majority of cells were the growing cells [8]. Other means by which persister cells can be eradicated were also investigated [12,20,21]. For example, Sultana et al. made an electrochemical scaffold (e-scaffold) that generates hydrogen peroxide (H_2_O_2_) to increase persister *Pseudomonas aeruginosa* PAO1 susceptibility to tobramycin in a biofilm [12]. The e-scaffold designed above completely eradicated persister cells with no evidence of viable cells remained [12]. According to the authors, the e-scaffold induced the generation of a hydroxyl radical (OH^−^) which increased the permeability of the cell membrane and killed the cells via oxidative damage [12]. Although the macroscale studies discussed above are interesting, they do not provide the mechanistic details by which persister cells differed in their phenotype from MDR cells treated at MIC or from untreated cells. Such fundamental mechanistic details are essential to designing effective antibiotic treatments capable of targeting persister cells based on their cellular properties. These mechanisms can be largely investigated using AFM. AFM studies in the literature which investigated how bacterial cells respond to antibiotics were limited to susceptible cells to antibiotics [77,79] and to cells resistant to antibiotics at MIC, including our own prior work [31,32]. For example, AFM was used to study the effect of ampicillin on the surface morphology [77,79,80], cell elasticity [77,79], and adhesion [49] of *E. coli* mostly using concentrations of antibiotics that are below MIC. Another study compared the effects of antibiotics on the cell membranes of susceptible and resistant *Pseudomonas aeruginosa* [76]. Their results demonstrate the abilities of antibiotics to disorganize the cell membrane at 4 mg/mL of ticarcillin, 0.25 mg/mL of tobramycin, and 32 mg/mL of tetra paraguanidinoethylcalix (CX1) [72]. To our knowledge, this study is the first AFM or nanoscale mechanistic study that investigated how persister cells respond to antibiotics in the literature. When all results of this study are combined, the following two mechanisms are proposed as a means by which persister cells resist ampicillin.

#### 2.8.1. Mechanism 1: Dormancy

Maintaining an appropriate cell shape is essential for the survival of persister cells. When cells keep an elliptical-like shape upon exposure to ampicillin, their small surface area to volume ratio is utilized to decrease their metabolic activities, conserve their energies, and avert their division, allowing them to go into dormancy [7,11,19,46]. Furthermore, we observed that the decrease in the cell surface area was directly proportional to the increase in membrane rigidity and in the grafting density of the surface biopolymers (Figure S7B). By increasing the volume and decreasing the bacterial surface area, persister cells may be decreasing their interactions with antibiotics while conserving their energies [37]. It may also be possible that the decrease in the surface area helps the cells to close their membrane pores and become more impermeable. It has been shown that many mutations which affect the synthesis of the bacterial cell wall PG are associated with changes in cell shape [81]. Changing from rod-like to less rod-like may alter the mechanism of PG insertion, turn off the elongation mode, and prevent β-lactams from accessing the PBPs [81].

#### 2.8.2. Mechanism 2: Increase in Cellular Elasticity

To increase cell elasticity, persister cells are expected to increase their surface roughness, collapse their surface biopolymers on their surfaces, and increase their apparent grafting densities. This indicates that, at long exposures to a high concentration of ampicillin (20× MIC), persister cells utilized their rigidity to resist ampicillin. Considering the link between cell shape and mechanical properties, it has been shown that, in some species, size reduction exhibited in non-dividing cells is a mechanism of survival [37]. The contraction of the cell membrane may change the molecular dynamics of the PG architecture [82] and possibly lead to a stiffer bacterial surface. In addition, upon the collapse of biopolymers on the bacterial surface, the likelihood of ampicillin meeting the PBPs is reduced. These findings likely explain why infections associated with persister cells are difficult to treat.

### 2.9. Implications of Our Findings on the Design of Effective Antibiotics against Persistent E. coli Cells

Administering antibiotics at MIC resulted in biofilm formation and at 20 times MIC resulted in more persister cells [31]. This finding points to an important problem that is faced when treating MDR infections. Therefore, development of new antibiotics should consider the complex properties of the bacterial surface. In this study, we showed that persister cells are stiffer than their resistant and untreated cells. Therefore, design of antibiotics that can break the stiffness of the cells and induce permeability should be considered. An example of success on that is the e-scaffold. Sultana et al. made an electrochemical scaffold (e-scaffold) that generates hydrogen peroxide (H_2_O_2_) to increase persister *Pseudomonas aeruginosa* PAO1 susceptibility to tobramycin in a biofilm [12]. This e-scaffold completely eradicated persister cells with no evidence of viable cells remaining [12]. According to the authors, the e-scaffold induced the generation of a hydroxyl radical (OH^−^) which increased the permeability of the cell membrane and killed the cells via oxidative damage [12]. Similarly, because of the possible reduction in permeability of cellular membranes, as cells were exposed to 20× MIC, design of small antibiotics capable of diffusing within the collapsed biopolymer structures of the bacterial surfaces should be a priority. 

## 3. Materials and Methods

### 3.1. Cells and Chemicals

Domestic multidrug-resistant *Escherichia coli* (MDR *E. coli*) Strains arbitrarily labeled as A5 and A9 were obtained from Prof. Douglas R. Call of the Paul G. Allen School of Global Animal Health, Washington State University. Ampicillin (100 mg/mL) and gelatin G2500-100G were acquired from Sigma-Aldrich, St. Louis, MO, USA. Luria–Bertani (LB) broth and agar were obtained from RPICorp, IL, USA. The LB broth was prepared by dissolving 10 g of NaCl, 5 g of yeast extract, and 10 g of tryptone into 1 L of deionized (DI) water per manufacturer’s instructions.

### 3.2. Choice of MDR E. coli as the Bacterial Model

The two Strains of MDR *E. coli* A5 and A9 were chosen because of their ability to form biofilms and their resistance to multiple antibiotics. The Strains were originally obtained from household samples in Tanzania under which they were exposed to water and were arbitrarily given the code names A5, and A9, respectively. In our previous publications [31,32], the means by which the physical characteristics of four Strains of MDR *E. coli* to resist ampicillin at or beyond minimum inhibitory concentration (MIC) were investigated. The two Strains investigated here were among the four previously studied. The characteristics previously explored were bacterial morphology, surface roughness, adhesion strength to a model surface of silicon nitride, biofilm formation, cellular elasticity, and the conformational properties of the bacterial surface biopolymers represented in terms of biopolymers’ brush thickness and grafting density.

Our previous studies [31,32] suggested two patterns with regards to how MDR *E. coli* cells resisted ampicillin at MIC. The first pattern involved cells increasing their surface area, enhancing their adhesion strengths to model surfaces and ability to form biofilms, elongating the thicknesses of their surface biopolymer brushes, and decreasing their elasticities [31,32]. With longer biopolymers and softer cellular membranes, cells were hypothesized to attach to surfaces well and to form stronger biofilms that enable them to impede the penetration of ampicillin within cells or biofilms [31]. The second pattern involved cells decreasing their dimensions and adhesion to surfaces, suggesting that cells go into dormancy [31]. For this group of MDR *E. coli* cells, a higher percentage of persister cells is to be expected [12,46,83,84]. Here, one MDR *E. coli* Strain representative of each pattern was selected to further explore their persistence means, especially at much higher MIC concentrations. Cells of *E. coli* Strain A9 represent the first pattern while cells of *E. coli* Strain A5 represent the second pattern discussed above.

The MICs of A5 and A9 were determined in our previous study to be 50 and 45 μg/mL, respectively [31]. Briefly, the guidelines of the Clinical and Laboratory Standard Institute for planktonic cultures were followed [85]. Per Strain, three individual colonies taken from three different LB plates were cultured in 5 mL LB at 37 °C and 150 rpm shaking for a day. Then, 100 μL of MDR *E. coli* cells were allowed to grow overnight each in the presence of a given concentration of ampicillin (0.2–400 μg/mL). Evidence of turbidity in a given test tube was indicative that cellular growth was not completely suppressed by the concentration of ampicillin used. The MIC was then taken as the lowest concentration of ampicillin required to prevent cellular growth. Once determined, newly cultured cells were challenged with the assessed MIC and no growth of cells overnight was used to confirm that the MIC quantified was accurate. Here, the cells were exposed to 20 times their MIC to induce persistence. The morphology, roughness, adhesion, elasticity, and conformations of bacterial surface biopolymers of persister cells isolated from Strains *E. coli* A5 and A9 were quantified and compared to those previously quantified by us for the resistant *E. coli* A5 and A9 cells [31,32].

### 3.3. Choice of the Model Antibiotic-Ampicillin

The choice of model antibiotic selected for this study was the β-lactam, ampicillin [31]. In this group of antibiotics, the basic structure consists of the β-lactam ring and a side chain [31]. The ring resides in the core structure and is responsible for the antibacterial activity through meddling of the function of Penicillin Binding Proteins (PBPs). PBPs are responsible for the synthesis of peptidoglycans (PGs) that form the rigid cell walls of bacteria such as *E. coli* [74]. The side chain of the β-lactam structure controls the range of antimicrobial activity as well as the compound’s pharmacokinetic properties [86]. Ampicillin is suitable for this study because its mode of action targets bacterial cell wall properties which can be easily probed with AFM [77,80]. AFM is a surface imaging technology, and thus is limited to characterizing changes that occur to the bacterial surface [77,80]. 

### 3.4. Bacterial Growth Conditions and Isolation of Persister Cells

For growth kinetics and persister cells’ isolation, two colonies of each MDR *E. coli* Strain from streak plates were cultured overnight in 5 mL LB broth each at 37 °C with continuous shaking at 220 rpm for a period of 12 h [87]. Subsequently, 0.5 mL each from the samples above were cultured in 50 mL of LB in a 125-mL round bottom culture Pyrex flask with continuous shaking at 220 rpm for 24 h until the optical density (OD) reached approximately 0.6 [8,87,88]. Cells were then collected by centrifugation at 16,000 *g* for 5 min and the pellets were resuspended in fresh 25 mL of LB without or with ampicillin at 20× MIC and incubated at 37 °C with continuous shaking at 220 rpm for a period of 25 h [87]. Cultures were grown in duplicates. One group of persister cultures was used for the AFM experiments and the second group was used to obtain the growth kinetics [87]. A negative control of untreated cells (no exposure to ampicillin) and a positive control of cells treated with ampicillin at MIC (resistant cells) were harvested after 3 h for AFM studies [31,32]. To obtain growth kinetics for persister cells, samples were left for 25 h and tested at time points, 0, 5, 10, 15, 20, and 25 h, respectively. For each time point, a 10-fold serial dilution of 0–10^−6^ was performed. A drop of a 10 µL of a bacterial suspension from each dilution was dropped onto an LB solid agar plate. The liquid suspension was spread on a large surface area for colonies to grow [87]. Three LB agar plates were incubated at 37 °C for a period of 24 h and the concentration of the colony forming units (CFU) per mL was determined for each plate [87].

### 3.5. AFM Experiments

All AFM measurements were performed in DI water with a Multimode AFM equipped with a Nanoscope IIIa controller and extender module (Bruker AXS Inc., Camarillo, CA, USA). Preceding any measurement, a 1 mL bacterial suspension cultured in LB in the presence or absence of ampicillin was pelleted by centrifugation at 5000 *g* for 5 min and washed three times with deionized water (DI) water. Three types of suspensions were pelleted. These were suspensions of untreated cells, those of cells treated at MIC that were harvested at OD of 0.5–0.7 (~800 × 10^3^ CFU/mL) (resister cells), and finally ones of persister cells treated at 20× MIC and harvested at an OD of ~0.1 corresponding to ~50 × 10^3^ and 30 × 10^3^ CFU/mL for Strains A5 and A9, respectively. Suspensions with bacterial cells taken from three different cultures and independent colonies were prepared. Each pellet was then resuspended in a 500 µL DI water. Then, 100 µL of the new suspension were dropped onto a fresh gelatin-coated mica surface prepared following our previously published protocol [31]. Bacterial cells were allowed to attach to the gelatin at room temperature for 10 min under DI water. Loosely attached bacterial cells were rinsed away from substrates with DI water. Per Strain and per treatment, three gelatin-coated mica substrates were prepared. For each Strain, 15 cells were probed per treatment. A triplicate was investigated adding up to 45 cells unless otherwise stated. Due to the expected heterogeneity of the bacterial surface, force measurements were made on locations that covered the entire bacterial surface. To do that, 25 points that spread over the entire bacterium were selected for measurements. To perform the AFM imaging and force measurements, silicon nitride (Si_3_N_4_) cantilevers were used (DNP-10, Bruker Inc., Santa Barbara, CA). These cantilevers were chosen for two reasons. First, they are hydrophilic [89]. Since ampicillin is hydrophilic [64] and forces are measured in the hydrophilic water, the adhesion forces measured would be expected to be significant and, as such, easy to be measured by AFM. Second, these cantilevers are easily available in the market. To calibrate the measured forces, the spring constant of each cantilever was determined prior to measurements using the method of the power spectral density of the thermal noise fluctuations in DI water [50,90]. On average, the spring constant was found to be 0.08 ± 0.03 N/m (*n* = 6), in good agreement with the manufacturer’s value of 0.06 N/m. The deflection sensitivity was measured on a cleaned mica surface in water and found to be 43.4 ± 5.7 nm/V (*n* = 6). All images of bacterial cells were captured at an average scan rate of 0.40 ± 0.04 Hz (*n* = 12) and at a resolution of 256 samples per line [31]. Since it is soft and can easily be damaged by the shear applied by the cantilever on cells upon contact, all force measurements on the bacterial surface were performed in tapping mode [91]. Here, approach curves were analyzed for cell elasticity, biopolymer brush thickness, and grafting density while retraction curves were analyzed for adhesion forces. When the cantilever contacts the biopolymers of the bacterial surface, a risk of contamination develops. To ensure that such contamination did not occur, force measurements on a clean mica background were always performed before and after measurements on bacteria. Equality of these forces measured between mica and the non-contaminated Si_3_N_4_ cantilevers confirmed that the tips were not contaminated. If measurements were different, a new tip was used [92].

### 3.6. Scanning Electron Microscopy (SEM) Imaging

To obtain the SEM images shown in Figure 3, cells were grown in LB supplemented with ampicillin at 45 or 50 µg/mL (MIC) for 3 h or in LB supplemented with ampicillin at 20× MIC for 25 h. Prior to imaging, cells were fixed with 2% paraformaldehyde and 2% glutaraldehyde in 0.1 M phosphate buffer at pH 7.2 overnight following a series of ethanol dehydration at 50%, 60%, 70%, 80%, and 2 × 100% [93,94,95]. Samples were finally dried in hexamethyldisilazane (HMDS) and sputter coated for 4 min to make a thin gold film of 7–10 nm 49–51. Images were captured with Tescan VEGA3 in a high vacuum mode using 30 kV.

### 3.7. Bacterial Morphology Analysis

Determining the morphologies of the bacterial cells and quantifying bacterial dimensions are crucial for understanding of many bacterial functions such as division and adhesion [96]. To assess bacterial morphology upon exposure to ampicillin, AFM height and phase images were captured concurrently in every single scan. Using the standard AFM Nanoscope Analysis 1.5 software (Bruker, Camarillo, CA, USA), the dimensions (length, width, and height) of individual bacterial cells were characterized [31]. The surface area (SA) and the surface area to volume ratio (SA/V) of individual cells were also estimated by assuming an elliptical cell geometry, as detailed in our prior publication [31].

### 3.8. Analysis of Bacterial Surface Roughness

The roughness of bacterial cell surface plays an important role in the cellular surface area available for adhesion [49]. The RMS values were determined over a constant area of 0.60 × 0.60 µm^2^ for each bacterium surface following the approach we detailed previously [31,47,97]. The small 0.36 µm^2^ area was selected to ensure that roughness was always assessed on the top of individual bacterial cells.

### 3.9. Analysis of Adhesion Forces

**Figure 9:**
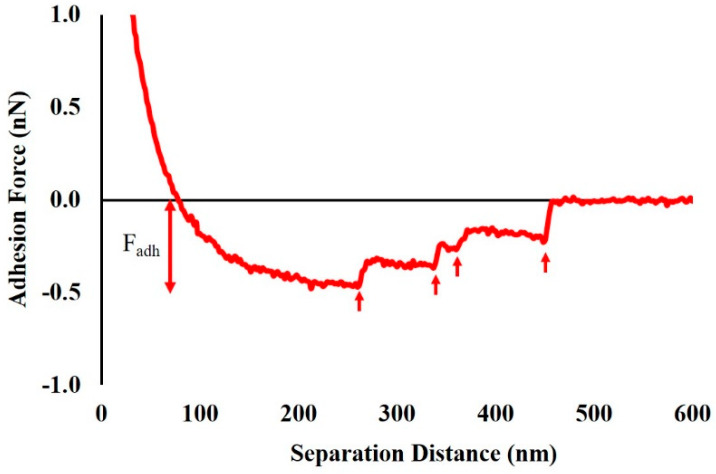
An example of a retraction force–distance curve measured between a Si_3_N_4_ cantilever and the surface biopolymers of *E. coli* Strain A5 in DI water under tapping mode in the absence of ampicillin. The adhesion peaks (F_adh_) on the retraction curve are indicated by red arrows.

Adhesion forces acting between the Si_3_N_4_ cantilever and the biopolymers of the bacterial surface in water were quantified using the AFM Nanoscope Analysis 1.5 software (Bruker, Camarillo, CA, USA) (Figure 9). Due to the expected heterogeneity among retraction curves, each curve was analyzed individually [98]. Example retraction curves for both Strains A5 and A9 are given in Figure S8. Data acquired from more than 1125 curves for each Strain were then compared among different ampicillin treatments and among the two Strains investigated.

### 3.10. Estimating the Length and the Grafting Density of a Bacterial Surface Biopolymer Brush Using the Steric Model

**Figure 10:**
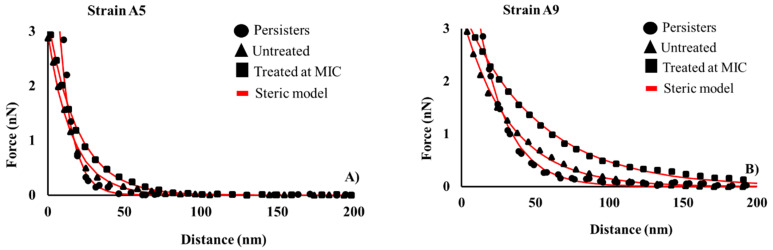
(**A**,**B**) Representative examples of how the steric model (red lines) fits the experimental data (symbols) for Strains A5 and A9 at different treatment conditions. The average *r^2^* for A5 was 0.95 and for A9 was 0.92.

The repulsive steric interaction forces expected between the negatively charged MDR *E. coli* cell surface biopolymers and the negatively charged model surface of the AFM Si_3_N_4_ cantilever and measured by AFM in the approach data were fitted to a steric model [91,99,100,101]. Steric interactions describe the conformational properties of the bacterial surface biopolymers and can be modeled by:

(1) $${F_{\mathrm{st}}=50K_{B}\mathrm{RT}L_{0}\Gamma }^{3/2}\mathrm{Exp}(-2\pi h/L_{0})$$

where *F_st_* is the steric force; *K_B_* is Boltzmann’s constant (1.3801 × 10^−23^ J/K); *T* is absolute temperature (298 K); *R* is the tip radius taken as 40 nm as measured by the manufacturer; *L*_0_ and *Γ* are the thickness (nm) and the grafting density (m^−2^) of the bacterial surface biopolymer, respectively; and *h* is the separation distance between the AFM tip and bacterium surface (nm). In Equation (1), *L*_0_ and *Γ* are the two fitting parameters [91,99]. Due to the heterogeneity in the approach force–distance data, each curve was fit to Equation (1) individually. Figure 10 shows examples of how the steric model fit the data for Strains A5 and A9 in the presence and absence of ampicillin.

### 3.11. Estimation of the Young’s Modulus of the Bacterial Cell

**Figure 11:**
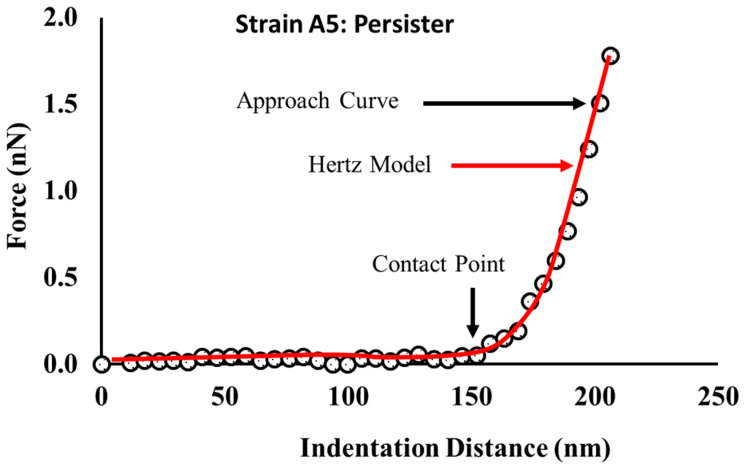
A representative indentation-force profile obtained on one of the A5 persister cells. The location of the contact point (CP) was obtained manually from the AFM Nanoscope Analysis 1.5 software (Bruker, Camarillo, CA, USA) and indicated by the downward black arrow. The force–indentation data were obtained from a persister cell exposed to a 1000 μg/mL of ampicillin for 25 h.

The Elastic modulus (E in MPa) was estimated from fitting the Hertz’s model of contact mechanics (Equation (2)) to the force–indentation data with a 1 nN maximum applied force (Figure 11).

(2) $$F\;=\frac{4}{3}\frac{E}{\left(1-\nu^{2}\right)}R^{1/2}\delta^{3/2}$$

According to the Hertz model, the force (F) required to indent a distance (δ) in the bacterial surface is a function of the non-deformable indenter radius (*R*, taken as 40 nm) and the Poisson ration ν taken as 0.5 for biological materials [92,102,103,104,105]. In Equation (2), *E* is the only fitting parameter and both F and δ are measured by AFM. Hertz model was fitted to the experimental data manually using the AFM Nanoscope Analysis 1.5 software (Bruker, Camarillo, CA, USA) [92,104].

### 3.12. Statistical Analysis

In this study, boxplots were used to represent the data because of their ability to showcase the heterogeneities expected in cellular properties in response to variable ampicillin treatments [11,50,66,67]. Boxplots display variations within a statistical population without making any assumptions of the underlying statistical distribution within the population [106]. The spacing between the different parts of the box plot reflect how many data contribute to each quartile and as such provide a quick visualization of the dispersion and skewness in data [106]. In addition to boxplots, histograms of all measured data were plotted and included in the Supplementary Materials as Figures S5–S8. Variations in cellular properties amongst untreated cells, resister cells, and persister cells were determined using One Way Analysis of Variance (ANOVA) available in Sigma Plot 11.0 (Systat Software Inc., San Jose, CA, USA). Because treatment groups were not always equal in size, Dunn’s test was used to compare the groups of interest. Statistical significance was considered at 99% confidence interval (*p* < 0.001).

## 4. Conclusions

Here, we demonstrated the potential of AFM to characterize the phenotypic morphological changes of MDR and persister cells in response to ampicillin at MIC and at 20× MIC, respectively. The phenotypic changes quantified were utilized to suggest mechanisms through which MDR and persister cells resist antibiotics. Our findings indicate that persister cells resist antibiotics by increasing their roughness, grafting densities of biopolymers, adhesion, and elasticities and by decreasing their surface areas to volumes ratio. We also observed that persister cells are heterogeneous. This heterogeneity can also be considered as a survival mechanism for exposed cells to antibiotics. Overall, our results underscore the importance of characterizing the roles of persister cells’ phenotypic means to resist ampicillin. Such better fundamental understanding is essential for the development of antibiotics capable of efficiently invading cell membranes and as such leading to eradication of persister cells.

## Supplementary Materials

The following are available online at https://www.mdpi.com/2079-6382/9/5/235/s1, Figure S1: The percentage survival of persister cells for Strains A5 (red line) and A9 (gray line and black symbols) over 25 hours period of exposure to ampicillin at 20× MIC, corresponding to 1000 and 900 μg/mL, respectively. Error bars reflect standard deviations from a triplicate, Table S1: Calculated cell dimensions for MDR-*E. coli* persister cells exposed to ampicillin at 20× MIC, resistant cells exposed to ampicillin at MIC and untreated cells. * Values are statistically significant from the untreated and treated groups. *^ Values are statistically significant from untreated cells but not from the treated groups, *p* < 0.001, *n* = 3 independent cultures. N is the number of cells characterized per group, Figure S2: Histograms displaying heterogeneities quantified in the adhesion force measured data between biopolymers of *E. coli* untreated (A and D), treated at MIC (B and E) and persister (C and F) cells for Strains A5 and A9, respectively. Solid lines are the normal distributions fits to the data. SD is standard deviation, Figure S3: Histograms displaying heterogeneities quantified in the estimated biopolymer brush thickness resulting from fitting the steric model (Equation (1)) to the approach distance-force data for *E. coli* untreated (A and D), treated at MIC (B and E) and persister (C and F) cells for Strains A5 and A9, respectively. Solid lines are the normal distributions fits to the data. SD is standard deviation, Figure S4: Histograms displaying heterogeneities quantified in the estimated biopolymer brush grafting density resulting from fitting the steric model (Equation (1)) to the approach distance-force data for *E. coli* untreated (A and D), treated at MIC (B and E) and persister (C and F) cells for Strains A5 and A9, respectively. Solid lines are the normal distributions fits to the data. SD is standard deviation, Figure S5: Histograms displaying heterogeneities quantified in the estimated Young’s modulus of elasticity resulting from fitting the Hertz model (Equation (2)) to the approach indentation-force data for *E. coli* untreated (A and D), treated at MIC (B and E) and persister (C and F) cells for Strains A5 and A9, respectively. Solid lines are the normal distributions fits to the data. SD is standard deviation, Figure S6: Scatter plots between bacterial polymer brush thickness and A) adhesion force and B) root mean square (RMS) roughness. Scatter plots between grafting density and C) adhesion force, D) root mean square (RMS) roughness, E) polymer brush thickness and F) elasticity, Figure S7: Scatter plots of bacterial surface area (SA) versus A) adhesion force, B) elasticity, and C) grafting density. D) Bacterial surface root-mean-square (RMS) roughness versus adhesion force. Data used were taken from both Strains, and Figure S8: Representative retraction curves of untreated cells, cells treated at MIC (resistant) and cells treated at 20× MIC (persisters) collected A) for Strain A5 and B) for Strain A9 in water.

## References

[1] Mohammad H., Mayhoub A.S., Cushman M., Seleem M.N. (2015). Anti-biofilm activity and synergism of novel thiazole compounds with glycopeptide antibiotics against multidrug-resistant *Staphylococci*. J. Antibiot. (Tokyo).

[2] Solomon S.L., Oliver K.B. (2014). Antibiotic resistance threats in the United States: Stepping back from the brink. Am. Fam. Physician.

[3] Aeschlimann J.R. (2003). The role of multidrug efflux pumps in the antibiotic resistance of *Pseudomonas aeruginosa* and other Gram-negative bacteria. Pharmacotherapy.

[4] Babic M., Hujer A.M., Bonomo R.A. (2006). What’s new in antibiotic resistance? Focus on beta-lactamases. Drug Resist. Update.

[5] Mohanty S., Mishra S., Jena P., Jacob B., Sarkar B., Sonawane A. (2012). An investigation on the antibacterial, cytotoxic, and antibiofilm efficacy of starch-stabilized silver nanoparticles. Nanomedicine.

[6] Read A.F., Woods R.J. (2014). Antibiotic resistance management. Evol. Med. Public Health.

[7] Lewis K. (2007). Persister cells, dormancy and infectious disease. Nat. Rev. Microbiol..

[8] Shah D., Zhang Z., Khodursky A., Kaldalu N., Kurg K., Lewis K. (2006). Persisters: A distinct physiological state of *E. coli*. BMC Microbiol..

[9] Bigger J.W. (1944). Treatment of infections with penicilin by intreatment sterilization. Lancet.

[10] Hobby G.L., Meyer K., Chaffee E. (1942). Observations on the mehanism of action of penicillin. Exp. Biol. Med..

[11] Gefen O., Balaban N.Q. (2009). The importance of being persistent: Heterogeneity of bacterial populations under antibiotic stress: Review article. FEMS Microbiol. Rev..

[12] Sultana S.T., Call D.R., Beyenal H. (2016). Eradication of *Pseudomonas aeruginosa* biofilms and persister cells using an electrochemical scaffold and enhanced antibiotic susceptibility. NPJ Biofilms Microbiomes.

[13] Amato S.M., Brynildsen M.P. (2014). Nutrient transitions are a source of persisters in *Escherichia coli* biofilms. PLoS ONE.

[14] Kim W., Conery A.L., Rajamuthiah R., Fuchs B.B., Ausubel F.M., Mylonakis E. (2015). Identification of an antimicrobial agent effective against methicillin-resistant *Staphylococcus aureus* persisters using a fluorescence-based screening strategy. PLoS ONE.

[15] Kwan B.W., Valenta J.A., Benedik M.J., Wood T.K. (2013). Arrested protein synthesis increases persister-like cell formation. Antimicrob. Agents Chemother..

[16] Lewis K. (2010). Persister Cells. Annu. Rev. Microbiol..

[17] Megaw J., Gilmore B.F. (2017). Archaeal persisters: Persister cell formation as a stress response in *Haloferax volcanii*. Front. Microbiol..

[18] Pu Y., Zhao Z., Li Y., Zou J., Ma Q., Zhao Y., Ke Y., Zhu Y., Chen H., Baker M.A.B. (2016). Enhanced efflux activity facilitates drug tolerance in dormant bacterial cells. Mol. Cell.

[19] Wood T.K., Knabel S.J., Kwan B.W. (2013). Bacterial persister cell formation and dormancy. Appl. Environ. Microbiol..

[20] Chowdhury N., Wood T.L., Martínez-Vázquez M., García-Contreras R., Wood T.K. (2016). DNA-crosslinker cisplatin eradicates bacterial persister cells. Biotechnol. Bioeng..

[21] Kwan B.W., Chowdhury N., Wood T.K. (2015). Combatting bacterial infections by killing persister cells with mitomycin C. Environ. Microbiol..

[22] Amato S.M., Orman M.A., Brynildsen M.P. (2013). Metabolic control of persister formation in *Escherichia coli*. Mol. Cell.

[23] Nguyen D., Joshi-Datar A., Lepine F., Bauerle E., Olakanmi O., Beer K., McKay G., Siehnel R., Schafhauser J., Wang Y. (2011). Active starvation responses mediate antibiotic tolerance in biofilms and nutrient-limited bacteria. Science.

[24] Williamson K.S., Richards L.A., Perez-Osorio A.C., Pitts B., Mclmmerney K., Stewart P.S., Franklin M.J. (2012). Heterogeneity in *Pseudomonas aeruginosa* biofilms includes expression of ribosome hibernation factors in the antibiotic-tolerant subpopulation and hypoxia-induced stress response in the metabolically active population. J. Bacteriol..

[25] Pamp S.J., Gjermansen M., Johansen H.K., Tolker-Nielsen T. (2008). Tolerance to the antimicrobial peptide colistin in *Pseudomonas aeruginosa* biofilms is linked to metabolically active cells, and depends on the pmr and mexAB-oprM genes. Mol. Microbiol..

[26] Moyed H.S., Bertrand K.P. (1983). hipA, a newly recognized gene of *Escherichia coli* K-12 that affects frequency of persistence after inhibition of murein synthesis. J. Bacteriol..

[27] Schumacher M.A., Piro K.M., Xu W., Hansen S., Lewis K., Brennan R.G. (2009). Molecular mechanisms of HipA-mediated multidrug tolerance and its neutralization by HipB. Science.

[28] Korch S.B., Henderson T.A., Hill T.M. (2003). Characterization of the hipA7 allele of *Escherichia coli* and evidence that high persistence is governed by (p)ppGpp synthesis. Mol. Microbiol..

[29] Korch S.B., Hill T.M. (2006). Ectopic overexpression of wild-type and mutant hipA genes in *Escherichia coli*: Effects on macromolecular synthesis and persister formation. J. Bacteriol..

[30] Keren I., Kaldalu N., Spoering A., Wang Y., Lewis K. (2004). Persister cells and tolerance to antimicrobials. FEMS Microbiol. Lett..

[31] Uzoechi S.C., Abu-Lail N.I. (2019). The effects of beta-Lactam antibiotics on surface modifications of multidrug-resistant *Escherichia coli*: A multiscale approach. Microsc. Microanal..

[32] Uzoechi S.C., Abu-lail N.I. (2019). Changes in cellular elasticities and conformational properties of bacterial surface biopolymers of multidrug-resistant *Escherichia coli* (MDR-*E. coli*) strains in response to ampicillin. Cell Surf..

[33] Keren I., Shah D., Spoering A., Kaldalu N., Lewis K. (2004). Specialized persister cells and the mechanism of multidrug tolerance in *Escherichia coli*. Bacteriology.

[34] Tamayo J., Humphris A.D.L., Owen R.J., Miles M.J. (2001). High-Q dynamic force microscopy in liquid and its application to living cells. Biophys. J..

[35] Beniac D.R., Siemens C.G., Wright C.J., Booth T.F.A. (2014). Filtration based technique for simultaneous SEM and TEM sample preparation for the rapid detection of pathogens. Viruses.

[36] Golding C.G., Lamboo L.L., Beniac D.R., Booth T.F. (2016). The scanning electron microscope in microbiology and diagnosis of infectious disease. Sci. Rep..

[37] Nyström T. (2004). Stationary-phase physiology. Annu. Rev. Microbiol..

[38] Justice S.S., Hunstad D.A., Cegelski L., Hultgren S.J. (2008). Morphological plasticity as a bacterial survival strategy. Nat. Rev. Microbiol..

[39] Si F., Li D., Cox S.E., Sauls J.T., Azizi O., Sou C., Schwartz A.B., Erickstad M.J., Jun Y., Li X. (2017). Invariance of initiation mass and predictability of cell size in Escherichia coli. Curr. Biol..

[40] Yoon M.Y., Lee K.M., Park Y., Yoon S.S. (2011). Contribution of cell elongation to the biofilm formation of *Pseudomonas aeruginosa* during anaerobic respiration. PLoS ONE.

[41] Young K.D. (2006). The selective value of bacterial shape. Microbiol. Mol. Biol. Rev..

[42] Young K.D. (2010). Bacterial shape: Two-dimensional questions and possibilities. Annu. Rev. Microbiol..

[43] Typas A., Banzhaf M., Gross C.A., Vollmer W. (2012). From the regulation of peptidoglycan synthesis to bacterial growth and morphology. Nat. Rev. Microbiol..

[44] Carballido-Lopez R. (2012). The actin-like MreB proteins in *Bacillus subtilis* a new turn. Front. Biosci..

[45] Zaritsky A., Woldringh C.L., Helmstetter C.E., Grover N.B. (1993). Dimensional rearrangement of *Escherichia coli* B/r cells during a nutritional shift-down. J. Gen. Microbiol..

[46] Spoering A.M.Y.L., Lewis K.I.M. (2001). Biofilms and planktonic cells of *Pseudomonas aeruginosa* have similar resistance to killing by antimicrobials. J. Bacteriol..

[47] Alves C.S., Melo M.N., Franquelim H.G., Ferre R., Planas M., Feliu L., Bardají E., Kowalczyk W., Andreu D., Santos N.C. (2010). *Escherichia coli* cell surface perturbation and disruption induced by antimicrobial peptides BP100 and pepR. J. Biol. Chem..

[48] Su H.N., Chen Z.H., Song X.Y., Chen X.L., Shi M., Zhou B.C., Zhao X., Zhang Y.Z. (2012). Antimicrobial peptide trichokonin VI-induced alterations in the morphological and nanomechanical properties of *Bacillus subtilis*. PLoS ONE.

[49] Laskowski D., Strzelecki J., Pawlak K., Dahm H., Balter A. (2018). Short communication effect of ampicillin on adhesive properties of bacteria examined by atomic force microscopy. Micron.

[50] Park B.J., Abu-Lail N.I. (2011). Atomic force microscopy investigations of heterogeneities in the adhesion energies measured between pathogenic and non-pathogenic *Listeria* species and silicon nitride as they correlate to virulence and adherence. Biofouling.

[51] Park B.J., Haines T., Abu-Lail N.I. (2009). A correlation between the virulence and the adhesion of *Listeria monocytogenes* to silicon nitride: An atomic force microscopy study. Colloid Surf. B.

[52] Berne C., Ducret A., Hardy G.G., Brun Y.V. (2015). Adhesins involved in attachment to abiotic surfaces by Gram-negative bacteria. Microbiol. Spectr..

[53] Donlan R.M. (2002). Biofilms: Microbial life on surfaces. Emerg. Infect. Dis..

[54] Garrett T.R., Bhakoo M., Zhang Z. (2008). Bacterial adhesion and biofilms on surfaces. Prog. Nat. Sci..

[55] O’Toole G., Kaplan H.B., Kolter R. (2000). Biofilm formation as microbial development. Annu. Rev. Microbiol..

[56] Vu B., Chen M., Crawford R.J., Ivanova E.P. (2009). Bacterial extracellular polysaccharides involved in biofilm formation. Molecules.

[57] Abu-Lail N.I., Camesano T.A. (2003). Role of ionic strength on the relationship of biopolymer conformation, DLVO contributions, and steric interactions to bioadhesion of *Pseudomonas putida* KT2442. Biomacromolecules.

[58] Limoli D.H., Jones C.J., Wozniak D.J., Cruz S. (2015). Bacterial extracellular polysaccharides in biofilm formation and function. Microbiol. Spectr..

[59] Sans-Serramitjana E., Fusté E., Martínez-Garriga B., Merlos A., Pastor M., Pedraz J.L., Esquisabel A., Bachiller D., Vinuesa T., Viñas M. (2016). Killing effect of nanoencapsulated colistin sulfate on *Pseudomonas aeruginosa* from cystic fibrosis patients. J. Cyst. Fibros..

[60] Falagas M.E., Kasiakou S.K. (2005). Colistin: The revival of polymyxins for the management of multidrug-resistant Gram-negative bacterial infections. Clin. Infect. Dis..

[61] Jacoby G.A., Medeiros A.A. (1991). More extended-spectrum beta-lactamases. Antimicrob. Agents Chemother..

[62] Thomson J.M., Bonomo R.A. (2005). The threat of antibiotic resistance in Gram-negative pathogenic bacteria: β-lactams in peril!. Curr. Opin. Microbiol..

[63] Eastman T., Zhu D.A. (1996). dhesion forces between surface-modified AFM tips and a mica surface. Langmuir.

[64] Nikaido H. (2003). Molecular basis of bacterial outer membrane permeability revisited. Microbiol. Mol. Biol. Rev..

[65] Renner L.D., Weibel D.B. (2011). Physiochemical regulation of biofilm formation. MRS Bull..

[66] Camesano T.A., Abu-Lail N.I. (2002). Heterogeneity in bacterial surface polysaccharides, probed on a single-molecule basis. Biomacromolecules.

[67] Van der Mei H.C., Busscher H.J. (2012). Bacterial cell surface heterogeneity: A pathogen’s disguise. PLoS Pathog..

[68] Zobell C.E. (1943). The effect of solid surfaces upon bacterial activity. J. Bacteriol..

[69] Gilbert P., Das J., Foley I. (1997). Biofilm Susceptibility to antimicrobials. Adv. Dent. Res..

[70] Stewart P.S. (2002). Mechanisms of antibiotic resistance in bacterial biofilms. Int. J. Med. Microbiol..

[71] Ramadan M.A., Tawfik A.F., Shibl A.M., Gemmell C.G. (1995). Post-antibiotic effect of azithromycin and erythromycin on streptococcal susceptibility to phagocytosis. J. Med. Microbiol..

[72] Tajkarimi M., Harrison S.H., Hung A.M., Graves J.L. (2016). Mechanobiology of antimicrobial resistant Escherichia coli and Listeria innocua. PLoS ONE.

[73] Vidya K., Mallya P., Rao P. (2005). Inhibition of bacterial adhesion by subinhibitory concentrations of antibiotics. Indian J. Med. Microbiol..

[74] Ghuysen J.-M. (1994). Molecular structures of penicillin-binding proteins and P-lactamases. Trends Microbiol..

[75] Vollmer W., Seligman S.J. (2010). Architecture of peptidoglycan: More data and more models. Trends Microbiol..

[76] Formosa C., Grare M., Jauvert E., Coutable A., Regnouf-de-Vains J.B., Mourer M., Duval R.E., Dague E. (2012). Nanoscale analysis of the effects of antibiotics and CX1 on a *Pseudomonas aeruginosa* multidrug-resistant strain. Sci. Rep..

[77] Perry C.C., Weatherly M., Beale T., Randriamahefa A. (2009). Atomic force microscopy study of the antimicrobial activity of aqueous garlic versus ampicillin against *Escherichia coli* and *Staphylococcus aureus*. J. Sci. Food Agric..

[78] Cerf A., Cau J.C., Vieu C., Dague E. (2009). Nanomechanical properties of dead or alive single-patterned bacteria. Langmuir.

[79] Longo G., Rio L.M., Trampuz A., Dietler G., Bizzini A., Kasas S. (2013). Antibiotic-induced modifications of the stiffness of bacterial membranes. J. Microbiol. Methods.

[80] Braga P.C., Ricci D. (1998). Atomic force microscopy: Application to investigation of *Escherichia coli* morphology before and after exposure to cefodizime. Antimicrob. Agents Chemother..

[81] Scheffers D.J., Pinho M.G. (2005). Bacterial cell wall synthesis: New insights from localization Studies. Microbiol. Mol. Biol. Rev..

[82] Nikaiido H., Pages J.M. (2012). Broad-specificity efflux pumps and their role in multidrug resistance of Gram-negative bacteria. FEMS Microbiol. Rev..

[83] Luby E., Ibekwe A.M., Zilles J., Pruden A. (2016). Molecular methods for assessment of antibiotic resistance in agricultural ecosystems: Prospects and challenges. J. Environ. Qual..

[84] Mokrozub V.V., Lazarenko L.M., Sichel L.M., Babenko L.P., Lytvyn P.M., Demchenko O.M., Melnichenko Y.O., Boyko N.V., Biavati B., DiGioia D. (2015). The role of beneficial bacteria wall elasticity in regulating innate immune response. EPMA J..

[85] Clinical Lab Standards Institute (2019). Performance Standards for Antimicrobial Susceptibility Testing.

[86] Filho R.P., Polli M.C., Filho S.B., Garcia M., Ferreira E.I. (2010). Prodrugs available on the Brazilian pharmaceutical market and their corresponding bioactivation pathways. Brazilian J. Pharm. Sci..

[87] Marques C.N.H. (2015). Isolation of persister cells from biofilm and planktonic populations of *Pseudomonas aeruginosa*. Bio-Protocol.

[88] Cañas-Duarte S.J., Restrepo S., Pedraza J.M. (2014). Novel protocol for persister cells isolation. PLoS ONE.

[89] Grant L.M., Ducker W.A. (1997). Effect of substrate hydrophobicity on surface−aggregate geometry: Zwitterionic and nonionic surfactants. J. Phys. Chem. B.

[90] Hutter J.L., Bechhoefer J. (1993). Calibration of atomic-force microscope tips. Rev. Sci. Instrum..

[91] Camesano T.A., Natan M.J., Logan b.E. (2000). Observation of changes in bacterial cell morphology using tapping mode atomic force microscopy. Langmuir.

[92] Park B., Abu-Lail N.I. (2010). Variations in the nanomechanical properties of virulent and avirulent *Listeria monocytogenes*. Soft Matter.

[93] Gomes L.C., Mergulhão F.J. (2017). SEM analysis of surface impact on biofilm antibiotic treatment SEM analysis of surface impact on biofilm antibiotic treatment. Scanning.

[94] Nishino T., Nakazawa S. (1976). 1033–1042 Bacteriological study on effects of beta lactam group antibiotics in high concentrations. Antimicrob. Agents Chemother..

[95] Thorn R.M.S., Greenman J. (2009). A novel in vitro flat-bed perfusion biofilm model for determining the potential antimicrobial efficacy of topical wound treatments. J. Appl. Microbiol..

[96] Young K.D. (2007). Bacterial morphology: Why have different shapes?. Curr. Opin. Microbiol..

[97] Girasole M., Pompeo G., Cricenti A., Congiu-Castellano A., Andreola F., Serafino A., Frazer B.H., Boumis G., Amiconi G. (2007). Roughness of the plasma membrane as an independent morphological parameter to study RBCs: A quantitative atomic force microscopy investigation. Biochim. Biophys. Acta-Biomembr..

[98] Park B.J., Abu-Lail N.I. (2011). The role of the pH conditions of growth on the bioadhesion of individual and lawns of pathogenic *Listeria monocytogenes* cells. J. Colloid Interface Sci..

[99] Abu-Lail N.I. (2003). The Effect of Biopolymer Properties on Bacterial Adhesion: An Atomic Force Microscopy (AFM) Study.

[100] Alexander S. (1977). Adsorption of chain molecules with a polar head a scaling description. J. Phys. Chem. B.

[101] Butt H.-J., Kappl M., Mueller H., Raiteri R., Meyer W., Rühe J. (1999). Steric forces measured with the atomic force microscope at various temperatures. Langmuir.

[102] Abu-Lail N.I., Camesano T.A. (2006). The effect of solvent polarity on the molecular surface properties and adhesion of *Escherichia coli*. Colloid Surf. B.

[103] Eskhan A.O., Abu-Lail N.I. (2013). Cellular and molecular investigations of the adhesion and mechanics of *Listeria monocytogenes* lineages’ I and II environmental and epidemic strains. J. Colloid Interface Sci..

[104] Ramezanian S., Uzoechi S.C., Muhunthan B., Abu-Lail N.I. (2018). Role of ionic strength in the thicknesses of the biopolymer fringes, spring constants, and Young’s moduli of *Pseudomonas putida*. J. Vac. Sci. Technol. B Nanotechnol. Microelectron..

[105] Vadillo-Rodriguez V., Schooling S.R., Dutcher J.R. (2009). In situ characterization of differences in the viscoelastic response of individual Gram-negative and Gram-positive bacterial cells. J. Bacteriol..

[106] Benjamini Y. (1988). Opening the box of a boxplot. Am. Stat..

