# Supplementary material for: Variations in the Morphology, Mechanics and Adhesion of Persister and Resister E. coli Cells in Response to Ampicillin: AFM Study
Source: Antibiotics (Basel). 2020 May 7;9(5):235. doi: 10.3390/antibiotics9050235 (PMC7277365; doi:10.3390/antibiotics9050235)
Supplement: Supplementary file 1 [file antibiotics-09-00235-s001.pdf]

Supplementary Materials for:

Variations in the Morphology, Mechanics and Adhesion of Persister and Resister *E. coli* Cells in Reponses to Ampicillin: AFM Study

Samuel C. Uzoechi[1,2] and Nehal I. Abu-Lail[3*]

[1]Gene and Linda Voiland School of Chemical Engineering and Bioengineering, Washington State University, Pullman, WA 99164-6515

[2]Department of Biomedical Technology, Federal University of Technology, Owerri, PMB 1526, Owerri, Nigeria

[3]Department of Biomedical Engineering and Chemical Engineering, The University of Texas at San Antonio, San Antonio, TX, 78249

[*]Corresponding Author:

Nehal I. Abu-Lail

Department of Biomedical Engineering and Chemical Engineering

The University of Texas at San Antonio

One UTSA Circle

AET 1.102

San Antonio, TX, 78249

210-458-8131

nehal.abu-lail@utsa.edu

Summary of Content

Number of Pages: 11

Number of Figures: 8

Number of Tables: 1

S.1. Cell viability as a function of ampicillin concentration and exposure time

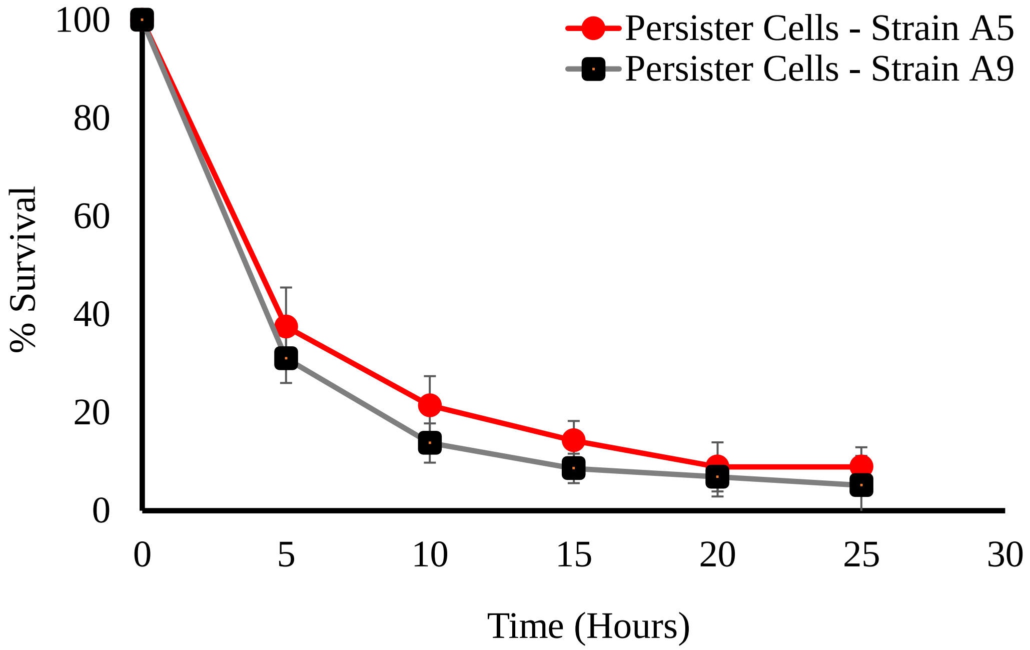

Figure S1: The percentage survival of persister cells for Strains A5 (red line) and A9 (gray line and black symbols) over 25 hours period of exposure to ampicillin at 20×MIC, corresponding to 1000 and 900 µg/ml, respectively. Error bars reflect standard deviations from a triplicate.

S.2. Bacterial morphology analysis

Table S1: Calculated cell dimensions for MDR-*E. coli* persister cells exposed to ampicillin at 20×MIC, resistant cells exposed to ampicillin at MIC and untreated cells. *Values are statistically significant from the untreated and treated groups. *^ Values are statistically significant from untreated cells but not from the treated groups, $p < 0.001$, n = 3 independent cultures. N is the number of cells characterized per group.

Strain ID	Treatment Condition	N	Width (μm)	Length (μm)	Height (μm)
MDR-*E. coli* A5	Untreated	43	1.9 ± 0.2	2.9 ± 0.6	0.7 ± 0.1
	Treated at MIC	42	1.8 ± 0.2*	2.6 ± 0.5*	1.0 ± 0.1*
	Persister cells	34	1.5 ± 0.2*	2.4 ± 0.5*^	0.8 ± 0.1*
MDR-*E. coli* A9	Untreated	45	1.5 ± 0.2	3.2 ± 0.4	0.6 ± 0.1
	Treated at MIC	15	2.3 ± 0.3*	13.2 ± 4.1*	0.8 ± 0.1*
	Persister cells	39	1.3 ± 0.1*	2.7 ± 0.5*	0.4 ± 0.1*

S.3. Heterogeneities in the measured adhesion forces between *E. coli* untreated, treated at MIC (resistant) and treated at 20×MIC (persistent) cells and silicon nitride in water.

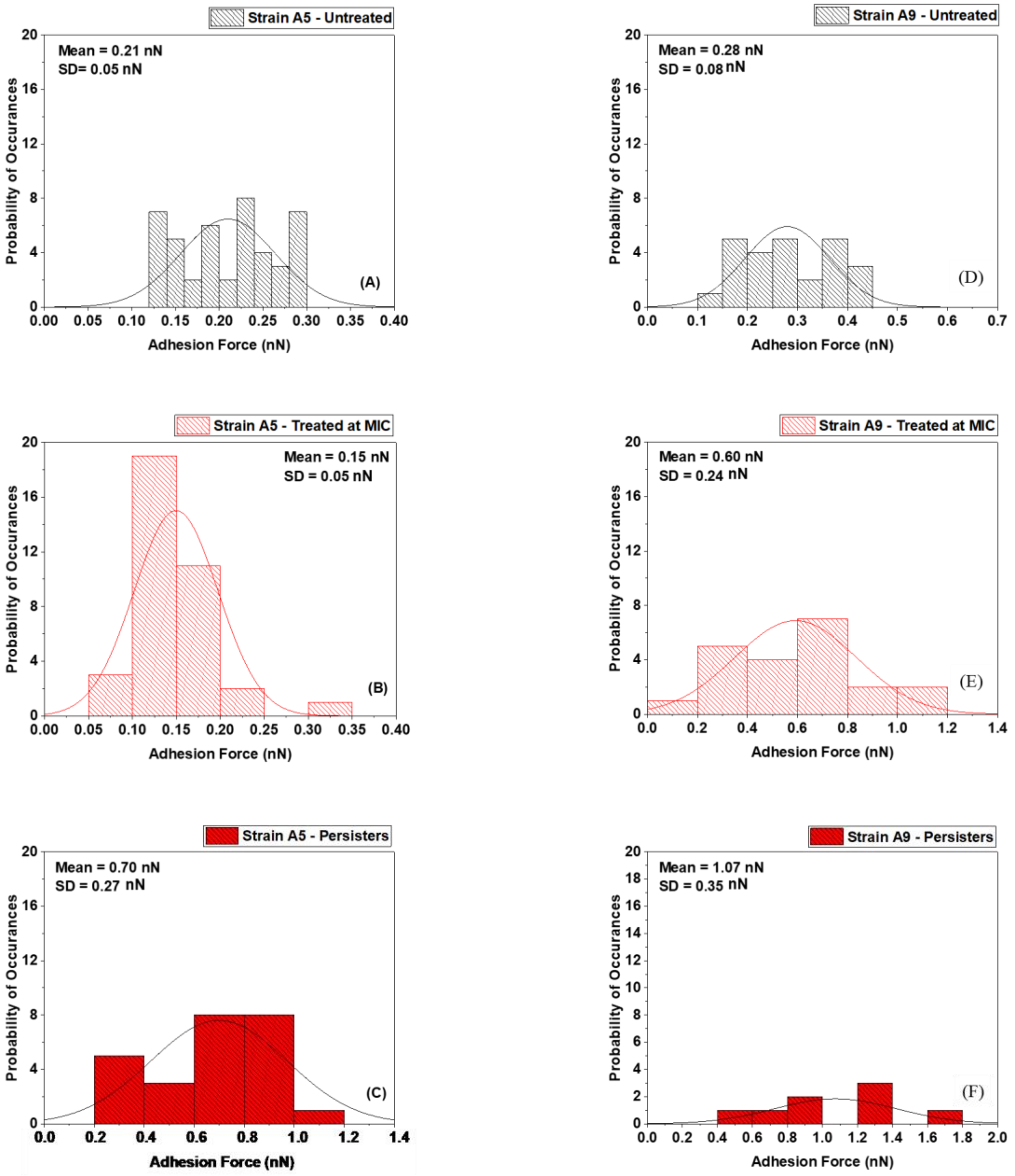

Figure S2: Histograms displaying heterogeneities quantified in the adhesion force measured data between biopolymers of *E. coli* untreated (A and D), treated at MIC (B and E) and persister (C and F) cells for Strains A5 and A9, respectively. Solid lines are the normal distributions fits to the data. SD is standard deviation.

S.4. Heterogeneities in the quantified biopolymer brush thickness for *E. coli* untreated, treated at MIC (resistant) and treated at 20×MIC (persistent) cells.

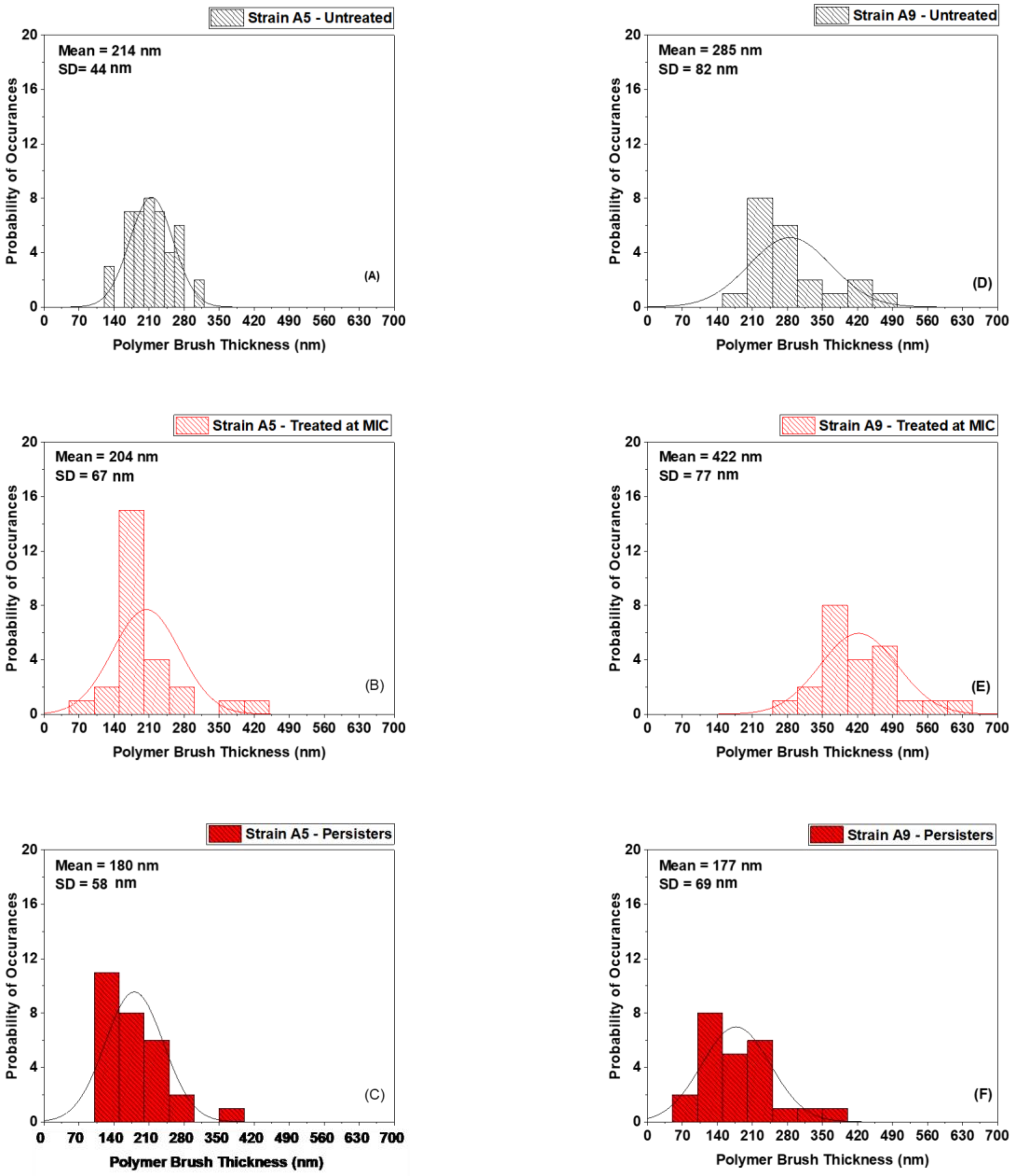

Figure S3**:** Histograms displaying heterogeneities quantified in the estimated biopolymer brush thickness resulting from fitting the steric model (eq. 1) to the approach distance-force data for *E. coli* untreated (A and D), treated at MIC (B and E) and persister (C and F) cells for Strains A5 and A9, respectively. Solid lines are the normal distributions fits to the data. SD is standard deviation.

S.5. Heterogeneities in the quantified biopolymer grafting density for *E. coli* untreated, treated at MIC (resistant) and treated at 20×MIC (persistent) cells.

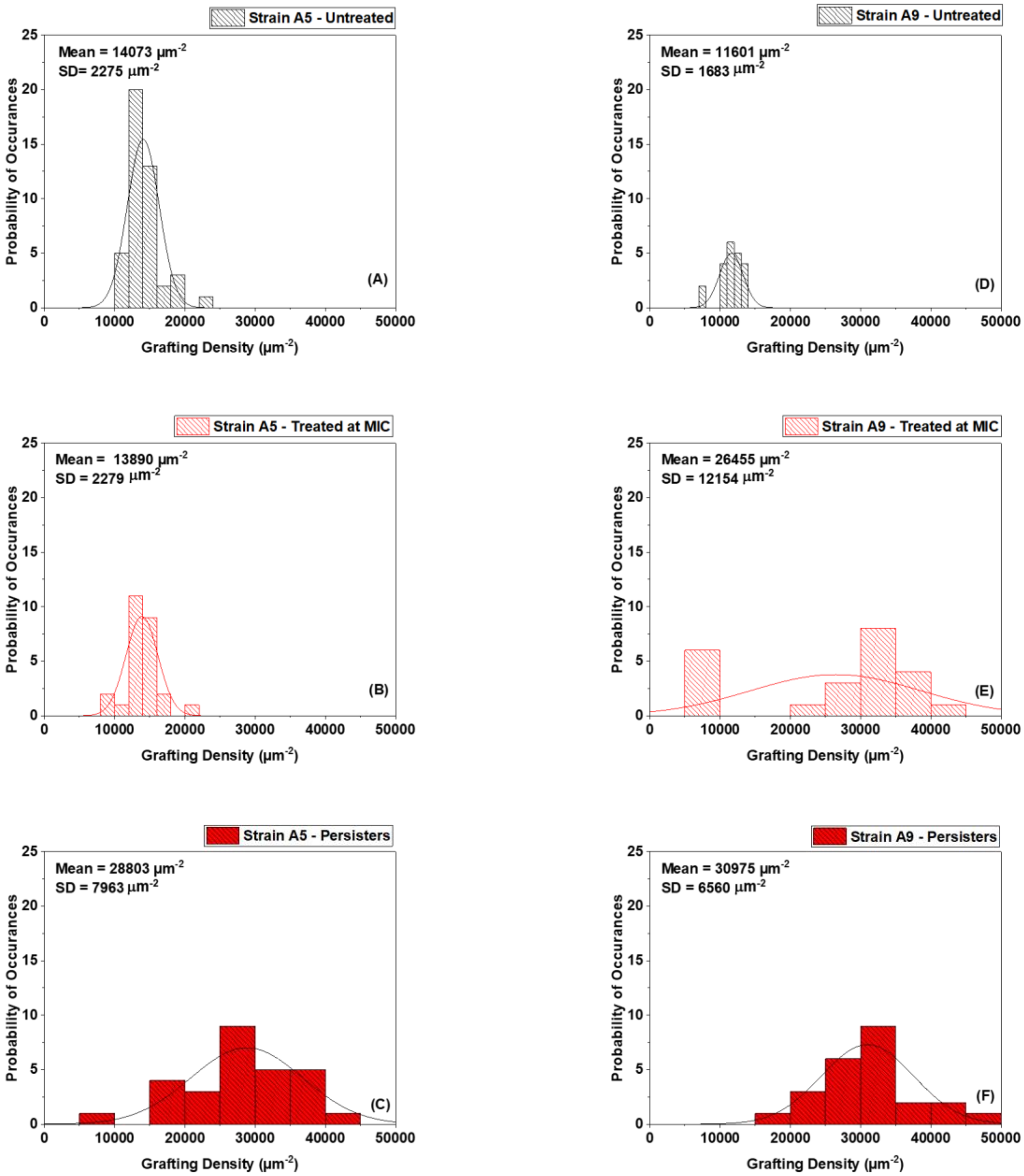

Figure S4: Histograms displaying heterogeneities quantified in the estimated biopolymer brush grafting density resulting from fitting the steric model (eq. 1) to the approach distance-force data for *E. coli* untreated (A and D), treated at MIC (B and E) and persister (C and F) cells for Strains A5 and A9, respectively. Solid lines are the normal distributions fits to the data. SD is standard deviation.

S.6. Heterogeneities in the quantified elasticities of *E. coli* untreated, treated at MIC (resistant) and treated at 20×MIC (persistent) cells.

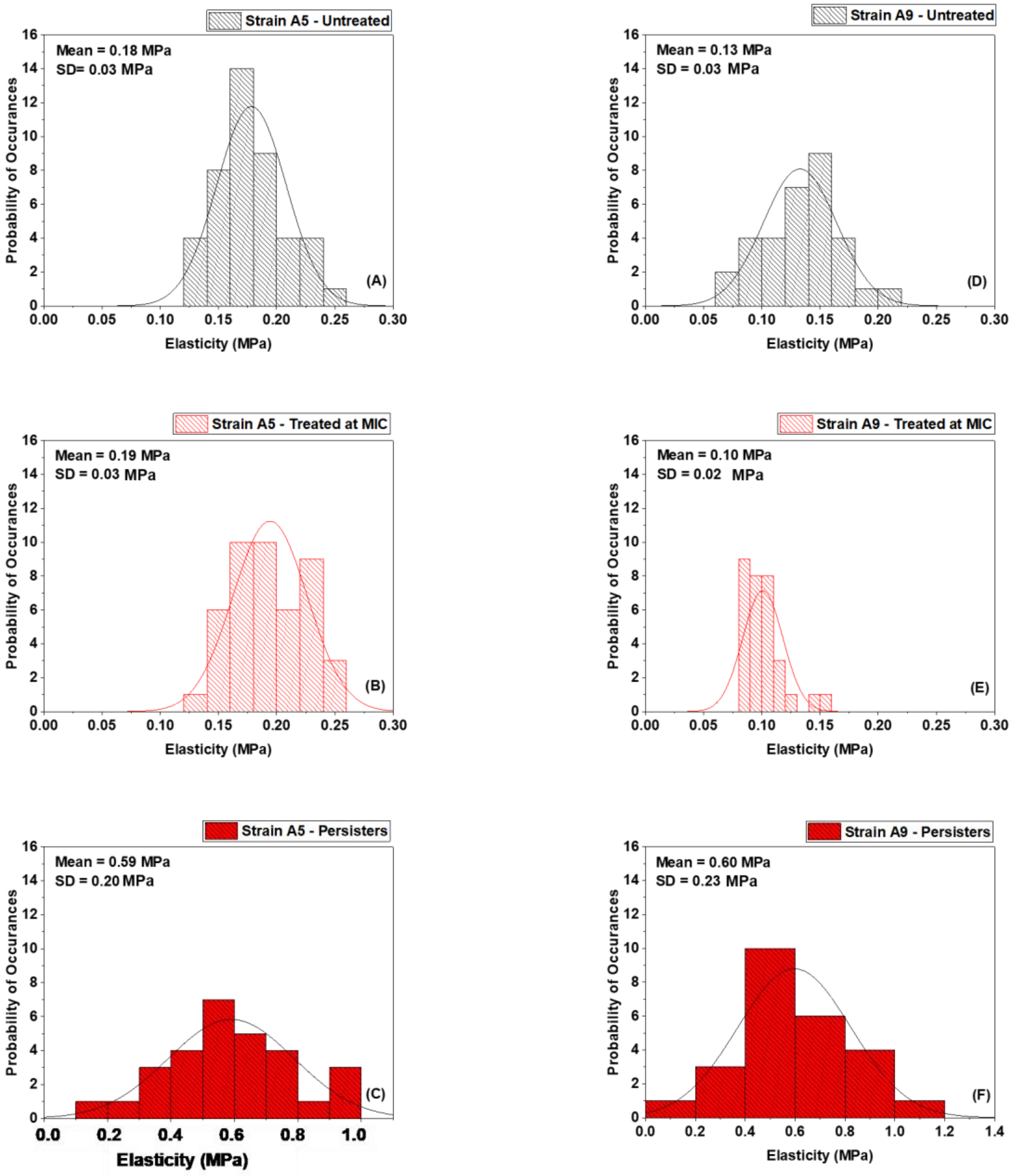

Figure S5: Histograms displaying heterogeneities quantified in the estimated Young’s modulus of elasticity resulting from fitting the Hertz model (eq. 2) to the approach indentation-force data for *E. coli* untreated (A and D), treated at MIC (B and E) and persister (C and F) cells for Strains A5 and A9, respectively. Solid lines are the normal distributions fits to the data. SD is standard deviation.

S.7. Possible relations amongst bacterial surface properties

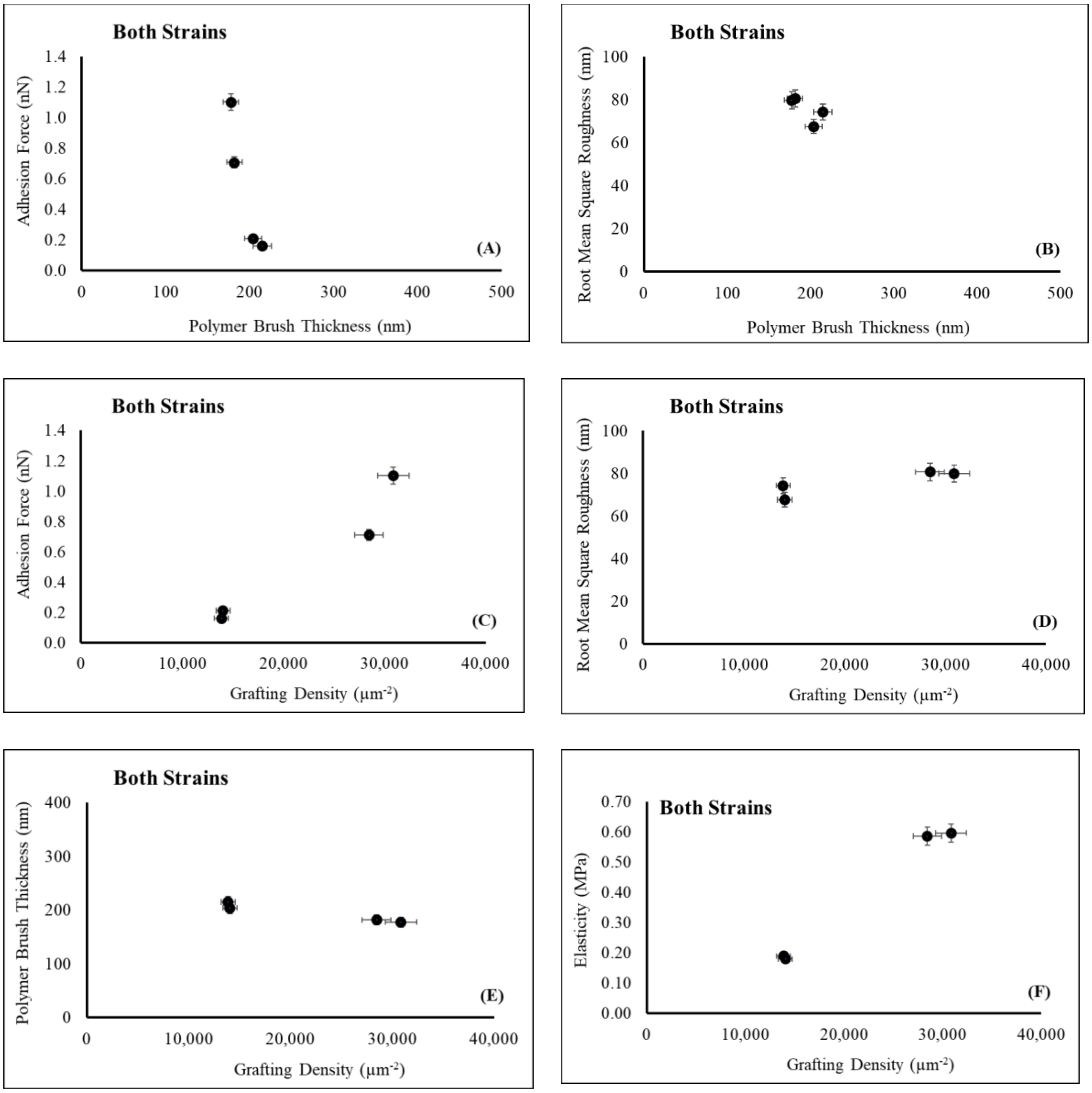

Figure S6: Scatter plots between bacterial polymer brush thickness and A) adhesion force and B) root mean square (RMS) roughness. Scatter plots between grafting density and C) adhesion force, D) root mean square (RMS) roughness, E) polymer brush thickness and F) elasticity.

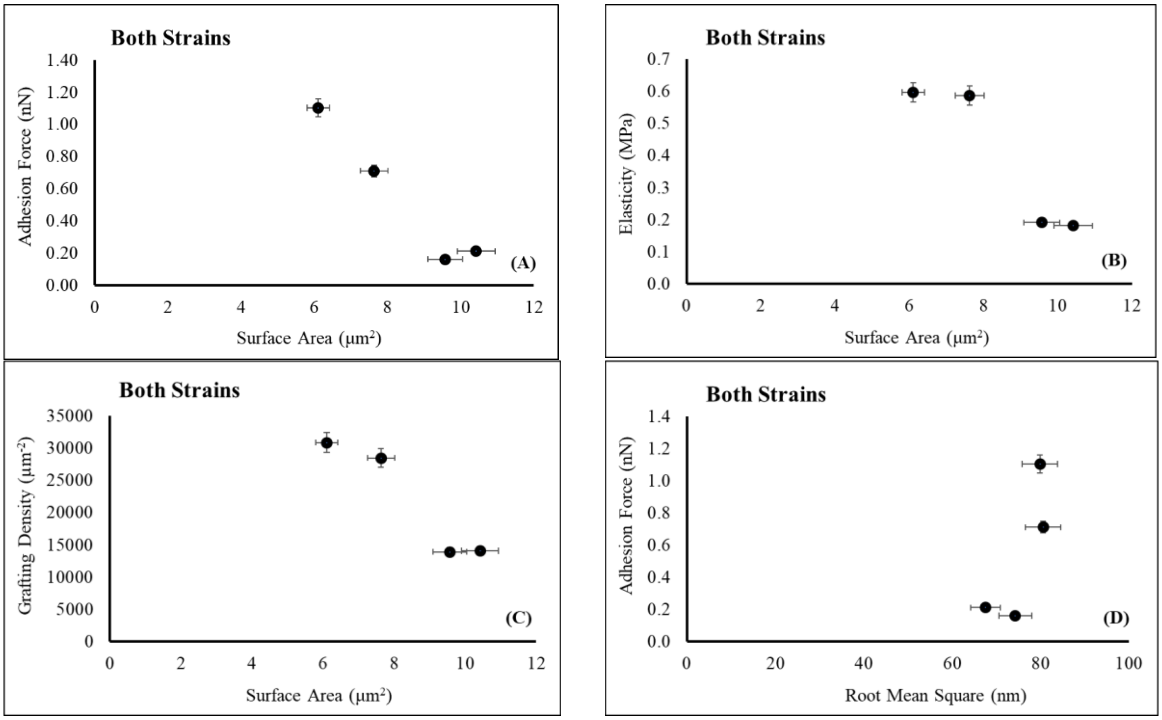

Figure S7: Scatter plots of bacterial surface area (SA) versus A) adhesion force, B) elasticity, and C) grafting density. D) Bacterial surface root-mean-square (RMS) roughness versus adhesion force. Data used were taken from both Strains.

S.8. Representative retraction curves for Strains A5 and A9

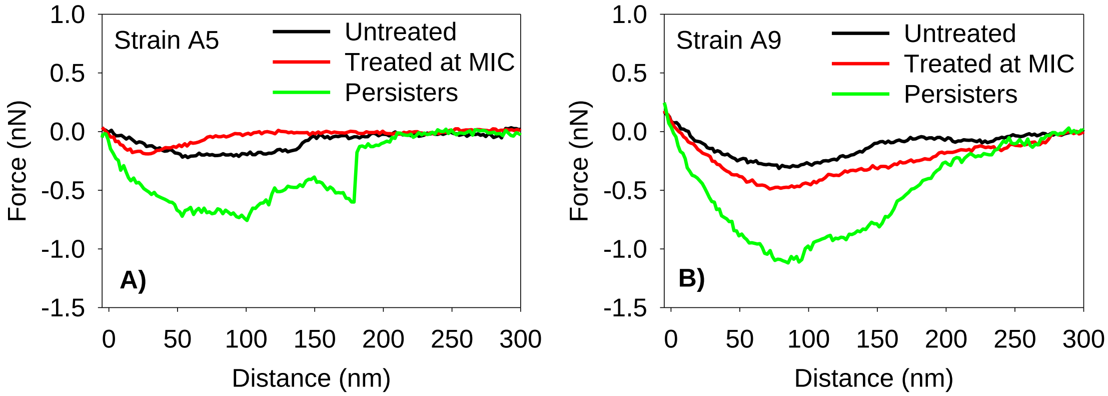

Figure S8: Representative retraction curves of untreated cells, cells treated at MIC (resistant) and cells treated at 20×MIC (persisters) collected A) for Strain A5 and B) for Strain A9 in water.
